# The controversial effect of smoking and nicotine in SARS-CoV-2 infection

**DOI:** 10.1186/s13223-023-00797-0

**Published:** 2023-06-01

**Authors:** Zahra Salehi, Bibi Fatemeh Nobakht Motlagh Ghoochani, Yazdan Hasani Nourian, Sadegh Azimzadeh Jamalkandi, Mostafa Ghanei

**Affiliations:** 1 Department of Immunology, School of Medicine, Tehran University of Medical Sciences, Tehran, Iran; 2 Chemical Injuries Research Center, Systems Biology and Poisonings Institute, Baqiyatallah University of Medical Sciences, Tehran, Iran

**Keywords:** Coronavirus disease, Nicotine, Smoke, Inflammatory diseases

## Abstract

The effects of nicotine and cigarette smoke in many diseases, notably COVID-19 infection, are being debated more frequently. The current basic data for COVID-19 is increasing and indicating the higher risk of COVID-19 infections in smokers due to the overexpression of corresponding host receptors to viral entry. However, current multi-national epidemiological reports indicate a lower incidence of COVID-19 disease in smokers. Current data indicates that smokers are more susceptible to some diseases and more protective of some other. Interestingly, nicotine is also reported to play a dual role, being both inflammatory and anti-inflammatory. In the present study, we tried to investigate the effect of pure nicotine on various cells involved in COVID-19 infection. We followed an organ-based systematic approach to decipher the effect of nicotine in damaged organs corresponding to COVID-19 pathogenesis (12 related diseases). Considering that the effects of nicotine and cigarette smoke are different from each other, it is necessary to be careful in generalizing the effects of nicotine and cigarette to each other in the conducted researches. The generalization and the undifferentiation of nicotine from smoke is a significant bias. Moreover, different doses of nicotine stimulate different effects (dose-dependent response). In addition to further assessing the role of nicotine in COVID-19 infection and any other cases, a clever assessment of underlying diseases should also be considered to achieve a guideline for health providers and a personalized approach to treatment.

**Implication**.

COVID-19 infection is currently the most prominent health challenge, and health decision-makers are looking for indicators to reduce the mortality and morbidity of the infection. Meanwhile, smokers make a considerable population, and current molecular data suggest a higher risk of COVID-19 infection due to the overexpression of Angiotensin-converting enzyme 2 (ACE2), the receptor for viral binding and entry. On the other hand, epidemiological studies show the controversial reports. According to previous investigations, we have systematically and comprehensively assessed nicotine’s effects on different cells and diseases, mainly in COVID-19. As the main result, we have recommended a research roadmap to identify a personalized approach for nicotine therapy (who, when, which dose, and how) for healthcare providers and researchers.

## Introduction

Coronaviruses are a large family of viruses in humans and animals, including Middle East Respiratory Syndrome (MERS-CoV), Severe Acute Respiratory Syndrome (SARS-CoV), and coronavirus disease 2019 (SARS-CoV-2 or COVID-19). Last two decades, several outbreaks of coronaviruses are observed. The SARS outbreak was reported in 2003 with about 8000 cases and more than 700 deaths with an estimated case fatality rate (CFR) of 10% [1]. According to WHO (Jan 2020), MERS has infected about 2500 cases with more than 800 deaths (CFR ≈ 34.3%) in different countries since 2012 [2]. The report of WHO showed the novel coronavirus pneumonia, COVID-19, has infected about 177,108,695 confirmed cases with 3,840,223 deaths until 18 June 2021 [3], and any protective or treatment factor is demanding for health managers.

Currently, there are contradictory reports about the effect of smoking on COVID-19 infections. If smokers are protective of COVID-19, new studies could be designed to explore the mechanism of protection. Nevertheless, if smokers are at higher risk, it will be a wake-up call for health providers to adopt separate policies to protect smokers. Currently, we know that smoking is one of the main risk factors for about 20 types and subtypes of cancers, cardiovascular diseases, Chronic Obstructive Pulmonary Disease (COPD), and diabetes. Among the various body parts, the most involvement is directly connected with the respiratory system, digestive system, and nervous system [4]. According to the 2025 estimation of the World Health Organization (WHO), there will be about 1.1 billion smokers worldwide [5]. International statistics show that there are about 8 million deaths each year, mostly (80%) in low- and middle-income countries, equivalent to a total economic cost of US$1.4 trillion each year [6].

Currently, it is clear that current and former smokers had a higher percentage among COVID-19 patients in ICU (~ 2.4 times) and mechanical ventilation support, and they had higher mortality rates and more severe cases (~ 1.4 times) [7–9]. Even after treatment, exacerbations of COVID-19 are associated with tobacco smoking in the smokers’ group [10]. Hospitalized smokers have more severe infections and complications, especially pulmonary fibrosis, as the central pathology of SARS, MERS, and COVID-19 [11]. Like MERS, a higher CFR in hospitalized patients with smoking history is also reported for COVID-19 [12].

Moreover, tobacco smoking is involved in more severe complications of COVID-19 infections [9]. It has been shown that cigarette smoke upregulates ACE2 in a dose-dependent manner in lung epithelium, including the goblet, club, Clara cells, and alveolar type 2 (AT2) cells of human and rodent lungs, and quitting smoking decreases ACE2 expression (reversible) [13]. So, smoking history is a predictor of ACE2 expression in the lung cells. The expression of Cathepsin B, which is a protease that activates the spike protein of the virus, is also increased in mice and human cells exposed to cigarette smoke [14]. So, it seems that the patients’ positive smoking history (current and former smokers) can make them more susceptible to COVID-19. Also, results regarding the effect of smoking on the expression of ACE2 are opposite; besides up-regulation (preprint) [15], down-regulation [16] is reported [17].

On the other hand, based on the current epidemiological studies on COVID-19, China [7, 18], Italia [19], Europe, and the United States [20] have reported lower incidence of infected smokers. Tajlil et al. had performed a meta-analysis on 12 reports of COVID-19 epidemiologic data (11,382 cases, including 3,827 cases in 10 Chinese reports and 7,555 cases in two USA reports). They had concluded the significantly lower proportion of hospitalized COVID-19 patients with smoking history [21]. It should be added that all the current smokers, when entering the hospital or the ICU, are forced to stop nicotine uptake. Besides, a large meta-analysis of over 17,278,392 COVID-19 infected adults showed a lower incidence of infection in smokers [22], which is concordant to former findings in China [7], France [23], and the United States [24]. However, several studies showed adverse effects of smoking on COVID-19 outcomes such as studies in Kuwait (1096 cases), England (3179 cases), and USA (12,347 cases) [25–27]. There is no solid evidence about the protective effect (prevention or treatment) of smoking on COVID-19 infection. The important thing to remember about epidemiological studies is that they have limitations, especially in this field. These limitations include things like the heterogeneity of COPD disease, the heterogeneity of COVID-19 disease, and the restrictions connected to it discussed the characteristics of epidemiological investigations, such as sample sizes and ascertainment bias [28].

In this study, we first tried to systematically show that cigarette smoke effects, although similar in some effects, are different from pure nicotine. Therefore, many experiments on the effect of smoke conclude their results as a probable effect of nicotine as an equivalent univalent. These conclusions can be misleading. Additionally, different cell responses to nicotine are dose-dependent. Considering the prevalence of the COVID-19 epidemic and different published results in this field and the existing judgments, an attempt was made to investigate the role of cigarettes and nicotine in this epidemic disease. Failure to assign this issue can cause misleading and diversion of people and health providers. Therefore, in this study, we have tried to take a closer basic-clinical look at this issue.

### Our approach and methodology

**Fig. 1:**
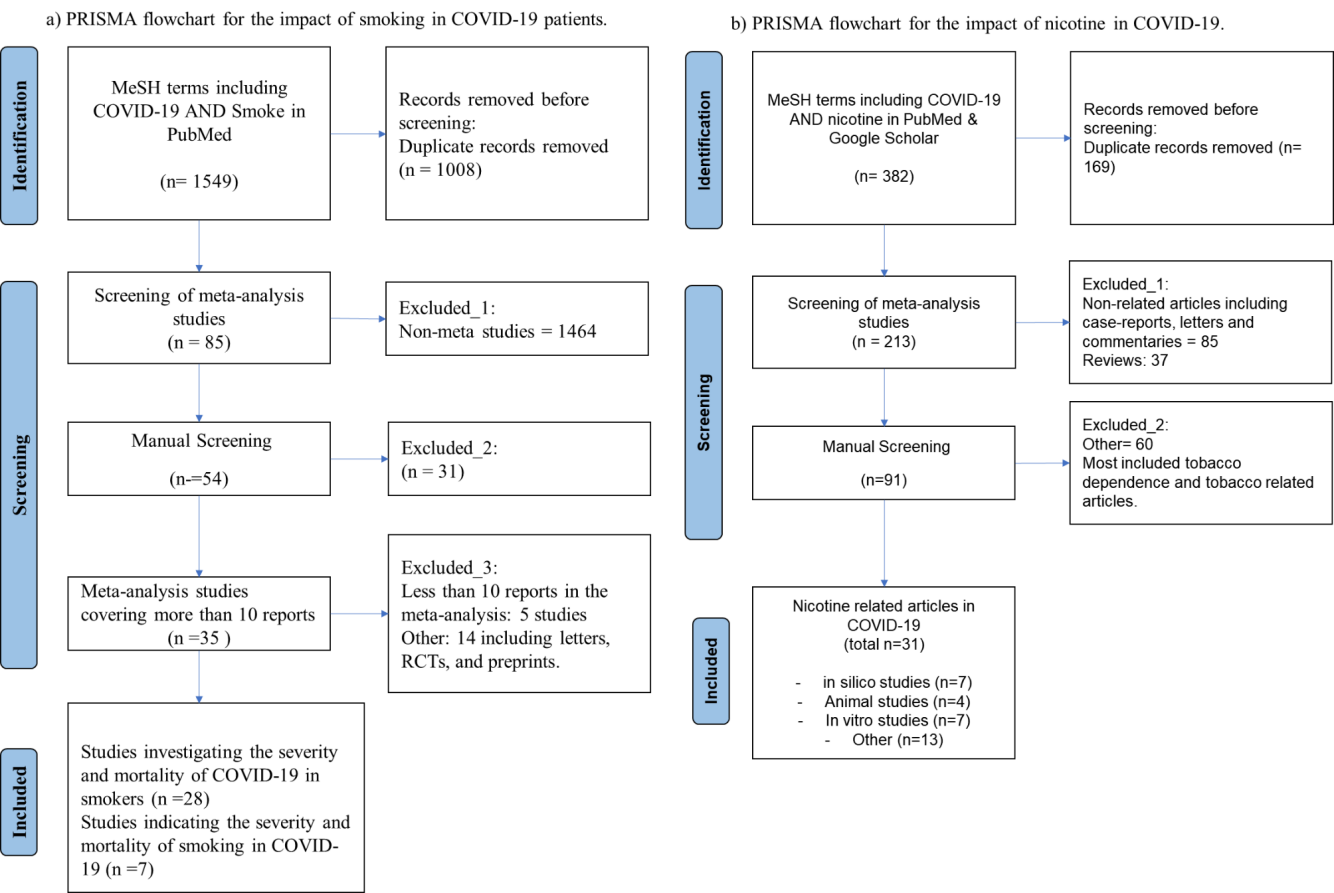
PRISMA table for (a) smoke and COVID-19 and (b) nicotine and COVID-19.

In the first step, articles containing the MeSH terms of “*smoke*” and “*COVID-19” and “meta-analysis”* were investigated only in PubMed (16 Jul 2021) (Fig. 1-a). Meta-analysis studies with more than 10 studies were summarized in Tables 1 and 2.

**Table 1:** The impact of smoking on the severity and mortality of COVID-19 patients in the different meta-analysis studies (> 10 studies)

Num	First author	Search history	Number of studies	Number of cases	Result	Ref.
1	Roengrudee Patanavanich	28 April 2020	19 papers (16 from China, 1 from Korea, and 2 from the United States)	11,590 patients:	Smoking is a risk factor for COVID-19 progression (OR 1.91).	[348]
2	Huimei Zhang	1 February 2021	109 articles (27 USA, 50 china, and 32 other countries)	517,020 patients	• Smoking elevated the risk of ICU admission and death in patients with COVID-19, but was not relevant to mechanical ventilation. • Former smokers had a risk of progressing COVID-19 severity compared with current smokers. Current smokers were significantly associated with the severity of COVID-19 compared with non-smokers.	[349]
3	Adinat Umnuaypornlert	12 December 2020	40 studies (19 China, 1 Kuwait, 1 Korea, 1 Mexico, 1 Japan, 2 Spain, 3 Italy, and 12 USA)	369,287 patients	Smoking, even current smoking or former smoking, significantly enhances the risk of COVID-19 severity and death.	[350]
4	Rohin K. Reddy	Between 1 December 2019 and 2 June 2020	47 studies (32 China, 10 USA, 2 Italy, 1 UK, 2 International)	32, 849 patients	Current smoking is risk factor for disease progression, severity and mortality in hospitalized patients with *COVID*-19.	[351]
5	Qianwen Zhao	Between December 2019 and 22 March 2020	11 studies (11 china)	2002 patients	COPD and current smoking could develop severity of COVID-19.	[352]
6	Tao Zhang	Between 1 January 2020 and 10 April 2020	16 studies (16 china)	1,172 severe patients and 2,803 non-severe patients	• The prevalence of former smokers was higher in severe patients as compared to non-severe ones. • The COVID-19 severity could be assessed by radiologic and laboratory findings, and smoking history	[353]
7	Linwen Zeng	Between 1 December 2019 and 2 May 2020	17 studies (16 China, 1 USA)	5,726 confirmed cases	Smoking did not enhance the risk of cardiovascular disease in COVID-19 patients in this study (probably due to small sample size, 3 studies, and large heterogeneity).	[354]
8	Jingyuan Xie	Between 1 January 2020 and 18 March 2020	90 studies (87 China, 1 Australia, 1 Singapore)	16,526 patients	Male gender, history of smoking, and comorbidities might affect the prognosis of COVID-19 patients.	[355]
9	Guiling Xiang	Between 1 December 2019 and 10 June 2020	20 studies	15,408 patients	Current smoking and elderly patients, aged 60 years old or over, are related with a higher risk of in-hospital death.	[356]
10	E. H. Taylor	Between 1 January 2020 and 6 December 2020	58 studies (15 China, 7 USA, 6 Spain, 3 UK, 2 Africa, 25 other countries)	44,305 patients	Increasing age, smoking, pre-existing comorbidities, and the host response to COVID-19 disease were associated with mortality.	[357]
11	David Simons	25 August 2020	32 studies	Not reported	In comparison with never smokers, current smokers seem to be at decreased risk of SARSCoV-2 infection whilst former smokers seem to be at elevated risk of hospitalisation, enhanced COVID-19 severity and mortality.	[358]
12	Angelo Silverio	27 April 2020	45 studies (35 China, 6 USA, and 4 other countries)	18,300 patients	• Male and smoking did not significantly influence mortality. • Older age and diabetes are related to greater risk of in-hospital mortality in COVID-19 patients	[359]
13	Saeed Shoar	Between 20 December 2019 and 15 March 2020	12 studies (12 China)	1,845 patients	Smoking and co-morbidities (hypertension, diabetes mellitus, cardiovascular diseases) are associated with COVID-19 mortality.	[360]
14	Changcheng Shi	Between 1 December 2019 and 29 April 2020	27 studies (24 China, 2 USA, and 1 Italy)	23,860 patients	Older age, sex (male), smoking (current smokers), pre-existing comorbidities (chronic kidney, respiratory, and cardio-cerebrovascular diseases), some symptoms (dyspnea), and some abnormal laboratory indicators (inflammation and coagulation markers) were associated with COVID-19 mortality.	[361]
15	Arthur Eumann Mesas	Between December 2019 and 27 July 2020	60 studies in 13 countries (31 China, 13 USA, 16 other countries)	51,225 patients	There was a greater mortality risk from hospital COVID-19 patients for dyspnoea (pooled OR = 2.5), smoking (pooled OR = 1.6) and some comorbidities and laboratory parameters.	[362]
16	You Li	Between January and May 2020	40 studies (18 China, 10 USA, 5 Italy, 7 other countries)	Not reported	• Male gender, older age, obesity, diabetes and chronic kidney diseases were associated with elevated risks for COVID-19 mortality. • There was no elevated risk of mortality for some factors such as COPD, cancer, or current smoker (probably because of limited data on every of these factors)	[363]
17	Xinyang Li	Between December 2019 and February 2021	41 studies (30 China, 3 Korea, 2 USA, 6 other countries)	21,060 patients	Severe COVID-19 patients were related to older age, male sex, obesity, history of smoking, hypertension, diabetes, coronary heart disease, chronic kidney disease (CKD), cerebrovascular disease, COPD, malignancy, and chronic liver disease.	[364]
18	Jia Li	Between December 2019 and 14 April 2020	12 studies (12 China)	2,445 patients	Smoking history and comorbidities such as COPD, diabetes, hypertension, coronary heart disease, cerebrovascular diseases, and malignancy were risk factors for severity of COVID-19.	[365]
19	Zohra S Lassi	Between December 2019 and February 2021	62 studies (from 44 countries of the six continents)	31,016 pregnant women	• Older pregnant women (> 35 years), obesity, smoking, diabetes and pre-eclampsia could increase the risk of severe COVID-19. • Severe COVID-19 women increased the risk of preterm birth.	[366]
20	Antonios Karanasos	Between 1 September 2019 and 4 May 2020	22 studies (20 China, 2 USA)	7,171 patients	The risk of severe COVID-19 was significantly greater among smokers, especially in younger patients without diabetes.	[367]
21	Shiwei Kang	Between 1 Jan and 6 October 2020	21 studies (17 China, 1 USA, 1 Japan, 1 England, 1 Italy)	7,041 patients	• Smoking history increased the mortality of COVID-19 patients (OR = 1.91). • Cardiovascular disease enhanced the severity (OR = 2.87) and mortality (OR = 3.05) of COVID-19 patients.	[368]
22	Ian Huang	25 March 2020.	23 studies (22 China and 1 Japan)	3,099 patients	• Age was associated with lymphopenia in COVID-19 patients (lymphopenia was higher in younger patients compared with older ones). • There was no association between lymphopenia and gender, cardiac comorbidity, hypertension, diabetes mellitus, COPD, and smoking.	[369]
23	Hongjie Hou	Between 15 January 2020 and 12 April 2021	73 articles (30 USA, 6 Italy, 7 England, 6 China, 6 Mexico, 4 Spain, and 14 other countries)	863,313 patients	Smoking elevated the mortality risk in COVID-19 patients.	[370]
24	Askin Gülsen	Between December 2019 and 15 April 2020	16 studies (14 China, 1 USA, 1 unknown)	11,322 patients	Current smoking was significantly associated with severe COVID-19.	[341]
25	Stefano Figliozzi	24 April 2020	49 studies (from China, Italy, Spain, France, Germany, Netherlands, Iran, and South Korea, USA)	587 790 and 602 234 cases statistics for age and sex.	• Older age, male gender, some co-morbidities such as acute cardiac or kidney injury, lymphocytopenia, and some laboratory biomarkers (D-dimer) elevated the risk of mortality in COVID-19 patients. • Smoking was not a predictor of the risk of death, it could increase adverse outcomes in COVID-19 patients.	[371]
26	Zhaohai Zheng	20 March 2020	13 studies (13 china)	3,027 patients	• The proportion of male, aged more than 65, smoking patients were statistically significant higher in critical/mortal group in comparison with the non-critical group.	[372]
27	Diana C. Sanchez-Ramirez	Between 1 January and 15 April 2020	22 studies (21 China and 1 USA)	13,184 patients	• The incidence of pulmonary diseases and smoking (current and former smoking) were significantly related to severe COVID-19 outcomes.	[373]
28	Alqahtani, J. S., et al. (2020)	March 24, 2020	15 studies (14 China, 1 United States)	2473 patients	• Increased severity (63%) and mortality (60%) in infected COPD patients. • Increased severity in infected current (22%) and ex-smokers (46%).	[342]

**Table 2:** Prevalence of smoking in COVID-19 patients in the different meta-analysis studies (> 10 studies)

Num	First author	Search history	Number of studies	Number of cases	Result	Ref.
1	Biruk Beletew Abate	Between 1 January 2020 and 27 March 2020	57 studies (52 china, 1 UK, 1 Italy, 1 Africa, 1 Japan, 1 Korea)	221,195 patients	A high prevalence of symptomatic COVID-19 was observed in men than women. One reason for the high prevalence of SARS-Cov-2 in men may be due to excessive cigarette and alcohol use in men.	[374]
2	Ashkan Baradaran	7 April 2020	33 studies (32 China and 1 Taiwan)	9,249 patients	The most prevalent finding in the confirmed patients with COVID-19 was hypertension, diabetes mellitus, cerebrovascular disease, cardiovascular disease, chronic kidney disease, chronic liver disease, chronic pulmonary disease, malignancy and **smoking of the patients**.	[375]
3	Jesus González-Rubio	28 April 2020	18 studies (15 China, 2 USA, and 1 Italy)	7,671 patients	The percentage of hospitalized current smokers was significantly lower than the smoking prevalence in each country.	[376]
4	Konstantinos Farsalinos	25 April 2020	30 studies (24 China, 4 USA, 1 South Korea, 1 Japan)	6,515 patients	• Low current smoking prevalence among hospitalized COVID-19 patients. • Odds of adverse outcomes were greater in hospitalized current smokers in comparison with non-current smokers’ • Odds of adverse outcomes were lower in hospitalized current smokers in comparison with former smokers.	[377]
5	Konstantinos Farsalinos	1 April 2020	13 studies (13 China)	5,960 patients	There was a low current smoking prevalence among hospitalized COVID-19 patients compared with smoking prevalence in the general Chinese papulation.	[378]
6	Kunchok Dorjee	31 August 2020	77 studies (35 China, 18 USA, 10 Europe, and 5 Asia)	38,906 patients	Smoking and several diseases such as hypertension, diabetes, and heart disease were higher prevalence among COVID-19 patients compared with the general USA population.	[379]
7	Francesco Del Sole	28 May 2020	12 studies (11 China and 1 Netherland)	2,794 patients	• Smoking, male gender and several diseases such as cerebrovascular disease, COPD, cardiovascular disease, diabetes, hypertension were related to severe disease. • Elevated level of some markers such as procalcitonin, D-Dimer and thrombocytopenia predicted severe of disease.	[380]

Also, articles containing the MeSH terms of “*nicotine*” and “*COVID-19*” were systematically searched in the online databases of PubMed, Google Scholar (Fig. 1-b). Then, all the articles were initially evaluated, and the preliminary design for the signaling and metabolic pathway involved in coronavirus infection and smoking/nicotine responses was performed. We performed a systematic search on (SARS-CoV-2 OR COVID-19) in PubMed and manually reviewed all the publications. Subsequently, all related articles were reviewed and included in this review (2021, June). Since the articles related to the study of the effect of nicotine in COVID-19 were very sparse, two other searching strategies regarding the effect of pure nicotine on different (A) cells, including epithelial, fibroblast, endothelial, and dendritic, macrophages, T, and B cells, and (2) organs, including respiratory, nervous, metabolic, cardiovascular, and urogenital systems. Generally, 12 diseases were candidate according to their similarity with the pulmonary and extrapulmonary complications of COVID-19. Also, Finally, the effect of smoking/nicotine on other common coronaviruses (SARS and MERS) was also evaluated and added to the study.

## COVID-19

**Fig. 2:**
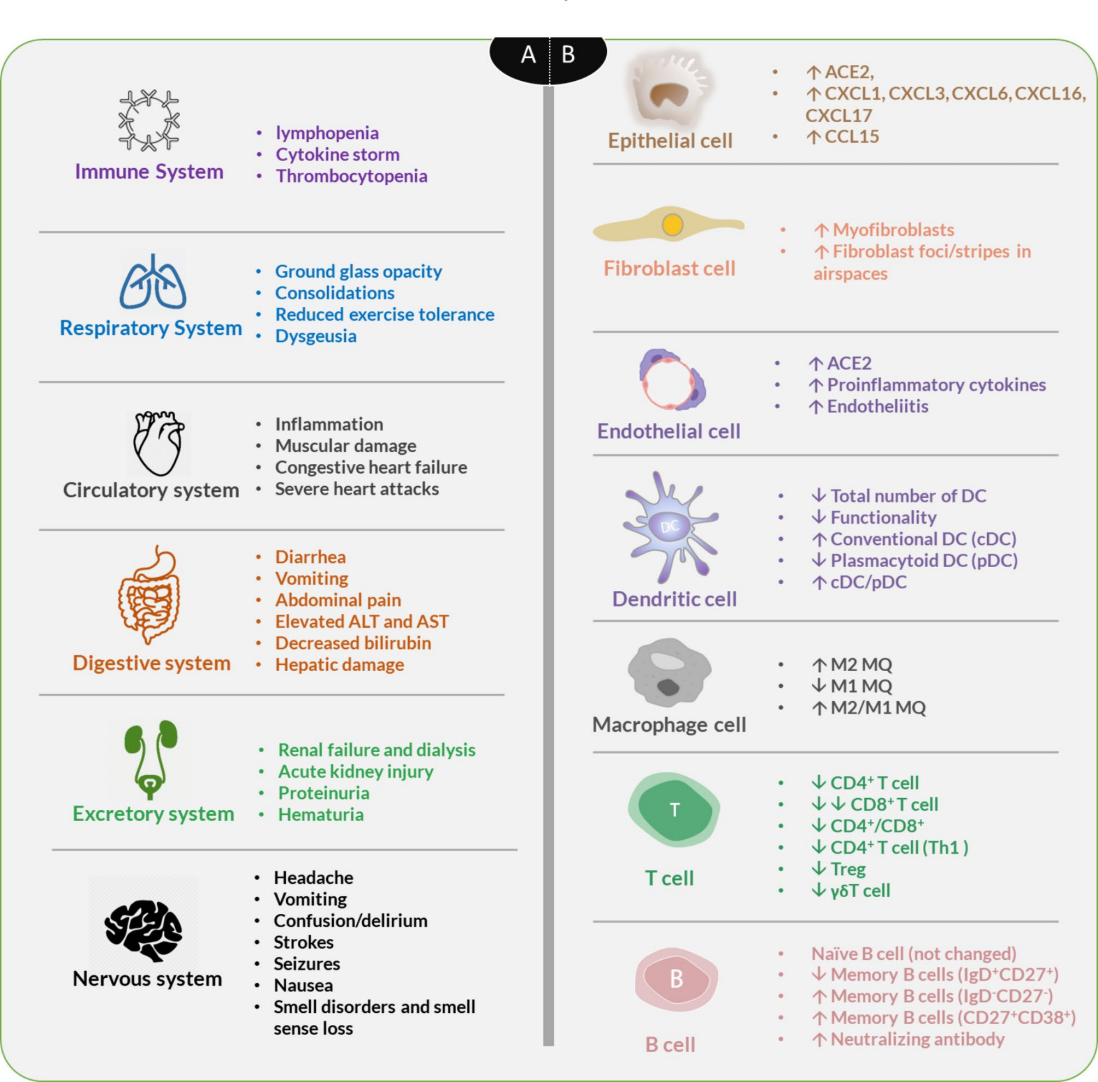
The multi-organ injury (A) and functioning of different cells (B) in SARS-CoV-2 infection

As a current health-threatening pandemic infection of the respiratory system, COVID-19 is accompanied by respiratory pneumonia symptoms (e.g., dry cough, fatigue, myalgia, fever, and dyspnea) and extrapulmonary symptoms mainly in the heart, liver, gut, kidney, and brain (Fig. 2) [29–31]. COVID-19 is an upper respiratory disease with systemic symptoms to distant organs/tissues due to distribution of diverse signaling molecules as a distant signaling mechanism. Chest tightness, breath shortness, and fatigue are severe symptoms of the disease. Patients with original comorbidities, including cardiovascular disease, cancer, kidney and liver diseases, diabetes mellitus, hypertension, tuberculosis, venous thromboembolism, and older adults, are high-risk cases for COVID-19 infection [32].

The renin-angiotensin system (RAS), which regulates water metabolism, endocrine secretion, and blood pressure in the body, is dysregulated during coronaviral infections. As an essential member of RAS, ACE2 is the common receptor for the attachment of SARS-CoV-2 and SARS-CoV. Coronaviruses identify a virus-binding hotspot region on ACE2 protein via spike protein [33]. ACE2 is a transmembrane protein that regulates the vascular system’s tuning and secretion of hormones within the RAS system, especially in the lung, heart, gastrointestinal, and kidney [34]. According to the current understanding, the expression of ACE2 was not different based on age, sex, or different racial groups [35]. Additionally, ACE2 downregulation after the SARS-CoV-2 infection is reported [36].

Of note, the ACE2 receptor is not adequate for viral entry into host cells. Recent evidence has shown that TMPRSS2 (transmembrane serine protease 2), a serine protease, may cleave the SARS-CoV-2 S protein [37]. Furthermore, the SARS-CoV-2 S protein has a furin cleavage site with the RPPA (Reverse Phase Protein Array) sequence [38]. Furin is a ubiquitous protease that is activated when exposed to an acidic pH [39]. Ubiquitous expressions of furin-like proteases may explain the increased pathogenesis of SARS-CoV-2. Studies have shown that RAS activation and ACE2 downregulation are implicated in the pathogenesis of lung damage following the SARS-CoV infection [40]. Renin, a protease secreted by the kidneys, converts angiotensinogen into angiotensin I (Ang I), which is metabolized by ACE into Ang II. Then Ang II is converted into Ang (1–7) by ACE2 [41]. SARS-CoV-2 causes downregulation of ACE2 because of enhanced levels of ACE2 shedding, which results in increased Ang II and reduced Ang (1–7) levels [40]. Ang II can bind to two classes of receptors, the Ang II receptor type 1 (AT1R) and Ang II receptor type 2 (AT2R) [42]. The binding of Ang II to AT1R activates protein kinase C (PKC), and induces pulmonary vasoconstriction and increased vascular permeability [43, 44]. PKC activation induces phosphorylation of the ADAM17 (ADAM Metallopeptidase Domain 17) tail and, subsequently, the phosphorylated ADAM17 tail enhances ACE2 shedding, assists with viral entry and tissue injury [45, 46]. PKC activation reduces amiloride-sensitive epithelial sodium channel (ENaC) expression, which is important for alveolar fluid clearance (AFC) and results in enhanced lung edema and worsened lung function [47]. Ji and colleagues reported that the S and E proteins of SARS-CoV diminish both activation and expression of ENaC by activating PKC isoforms [48]. ENaC-ɑ must be activated by proteases, particularly furin, in order for it to function. Anand et al. reported that SARS-CoV-2 hijacks proteases and these proteases subsequently activate ENaC-ɑ for viral activation. Thus, reduced ENaC function could be a reason for manifested pulmonary edema in COVID-19 infections [49]. Besides, activation of PKC downregulates AT2R gene expression [50]. Ang II via AT2R has the opposite effect, it activates the bradykinin-nitric oxide (NO)-cGMP cascade, and, subsequently, vasodilation occurs. Notably, Ang II and Ang (1–7) have the greatest relative affinities for AT1R and AT2R [42].

Besides, most of the patients with COVID-19 show lymphopenia (low level of lymphocytes) and hypercytokinemia (cytokine storm), which is intensified in ICU than non-ICU patients were also evident in SARS-CoV cases in 2003. These two dysregulated systemic responses are associated with increased severity and mortality of the patients [51]. Most of the patients with lymphopenia contained reactive lymphocytes, which was not observed in 2003 epidemy. After the viral entrance, the absolute number of T-lymphocytes and the frequency of CD4^+^ and CD8^+^ T cells, and the CD4^+^/CD8^+^ ratio are decreased in COVID-19 patients. Also, interferon-gamma )IFNγ( producing CD4^+^ T cells from severe COVID-19 patients are lower than moderate cases [52]. In COVID-19 patients, M2 macrophages dominate M1 types in BAL samples of cases, indicating their crucial involvement in inflammation and fibrogenesis [53]. The serum amount of IL1, IL6, IL8, IL10, and IL2R, and high-sensitivity C-reactive protein (hs-CRP) are upregulated in cases at the admission. Also, ICU cases express more cytokines than non-ICU patients [53]. SARS-CoV nucleocapsid protein (N protein) activates IL6 expression in a concentration-dependent manner, which requires NF-κB binding cis-acting element at the IL6 promoter region [54]. Further, SARS-CoV activates NLRP3 inflammasome, which upregulates the expression of IL1β and IL18 proinflammatory cytokines in lipopolysaccharide (LPS)-primed macrophages via 3a protein (also known as 3a-NLRP3-IL1b) signaling axis [55]. Generally, the platelet count is normal in COVID-19 patients, but some patients show mild thrombocytopenia (low platelets) [18]. Endothelial dysfunction and organ failure are associated with SARS-CoV-2 infections [56] (Fig. 2). The previous evidence showed that increased levels of pro-inflammatory cytokines may promote hypercoagulative factors, which induce vascular thrombosis [57, 58]. ACE2 down-regulation might be the other etiology of hypercoagulable state in patients with COVID-19, it is a negative feedback regulator of ACE2 expression via activating MAPK1 and MAPK3. Besides, elevated level of Ang II or ACE/Ang II/AT1 axis after ACE2 down-regulation resulting in extreme pro-inflammatory and pro-thrombotic cytokines [57, 59].

## Nicotine

### Nicotine

Tobacco constitutes a varied mixture of > 8000 chemicals, including nicotine, carbon monoxide, nitrogen oxides, pro-oxidants (e.g. free radicals), aromatic amines, catechols, inorganics (e.g. nickel, chromium, and cadmium) [60, 61]. Although the exact mechanisms by which smoking causes diseases are unclear, chronic inflammation is the main factor in the development of diseases relevant to smoking such as lung cancer, heart disease, COPD, and asthma [61, 62]. Nicotine (*C*_*10*_*H*_*14*_*N*_*2*_), a potent parasympathomimetic alkaloid, is the primary addictive tobacco smoke component. It was named after Jean Nicot, the French ambassador to Portugal, who introduced tobacco seed in Paris in 1550 [63]. A single cigarette tar contains about 10 to 12 mg nicotine, and when a cigarette is smoked, between 1 and 2 mg is inhaled into the lungs [64]. Nicotine interferes with the natural functioning of acetylcholine and over-stimulates acetylcholine receptors. In addition to the activation of nicotinic receptors, nicotine can be absorbed from cellular membranes, especially through the epithelium of the lower respiratory system and alveolus [65]. After absorption through the lungs, nicotine is metabolized in the liver by CYP2A6, UDP glucuronosyltransferase (UGT), and flavin-containing monooxygenase (FMO) enzymes. Cotinine is the principal (70–80%) product of nicotine metabolization and is used as a biomarker in smokers, second-hand smokers, and children exposed to smoke (detectable in blood, urine, saliva, hair, and nail) [66, 67].

Nicotine is only one of 8,000 tobacco components in cigarettes; therefore the consequences of smoking cannot be attributed only to nicotine [68]. In one study, the cytotoxicity of cigarette smoke extract (CSE) containing nicotine (CSE-N) and nicotine-free portion (CSE-O) on human bronchial epithelial cells was studied (BEAS-2B). CSE and CSE-O were shown to be hazardous to BEAS-2B cells, but CSE-N exhibited substantially lower cytotoxicity. CSE-O, but not CSE or CSE-N, enhanced apoptosis in cells stably expressing CYP2A13 (B-2A13). Cytochrome P450 2A13 (CYP2A13), an extrahepatic enzyme mostly expressed in the human respiratory system, has been discovered to mediate cigarette smoke metabolism and toxicity. These findings show that the nicotine component reduces the metabolic activation of CYP2A13 to CSE. As a result, it may be useful in lowering the toxicity produced by other tobacco components [69]. In fact, much more research is required to establish whether pure nicotine has detrimental or good effects, although it can be stated that many of the consequences induced by cigarette smoke, particularly in respiratory problems, are caused by other components in cigarette smoke.

#### Nicotine-responsive receptors

Nicotine imitates the function of acetylcholine, the principal neurotransmitter in the central nervous system (CNS), by binding to the various acetylcholine receptors. Acetylcholine can bind to two different receptors, including nicotinic (nAChRs) and muscarinic (mAChRs) cholinergic receptors, activated by specific nicotine and muscarine agonists, respectively. The competitive binding of nicotine to nicotinic cholinergic receptors induces a rapid transient allosteric conformational change to form a selective ionic channel. Activation of the receptors in the CNS activates dopamine, the pleasure hormone [70].

After stimulation of the cells with nicotine, calcium internalization increases (~ 100 fold), associated with loss of membrane integrity [71]. Also, in the case of continuous exposure of the receptor to an agonist, long-term inactivation happens. The constant stimulation of the receptor downregulates its expression to provide the tolerance effect [72]. Therefore, smokers need to increase their smoke to take a constant effect. Prolonged nicotine consumption/smoking reduces active receptors (unstimulated receptors) and dopamine release that stimulates the increase of smoking packs to recover their pleasure [72]. After a sleeping period, nicotine concentration is decreased, and more receptors are in the unoccupied status, and more dopamine is accumulated within the cells, which can induce maximal pleasure during the first-smoke of the day. Inactivation of the receptors and their downregulation and decreased dopamine release is called tolerance, which makes the dependency on smokers and addiction [73].

The nAChRs are divided into two different groups of neuronal and non-neuronal receptors based on their expression in various tissues. Structurally, this receptor family is a homo(hetero) pentamer composed of a combination of α(1–10), β(1–5), ε/γ, and δ subunits [74]. There is a specific neuronal and non-neuronal pentamer combination of subunits distributing throughout the body. Among them, α7 pentamer is the most studies homo-pentamer, which interacts with nicotine [75]. Non-neuronal nAChRs are expressed in lung epithelial, endothelial, fibroblast cells, and also in muscles. Seemingly, bronchial epithelial cells express more muscle-type nAChRs [76, 77]. Also, fibroblasts are enriched for more nAChRs subunits than epithelial cells.

There are many publications investigating the interplay between nicotine and nAChRs [78, 79]. Nicotine exposure changes the expression of nAChRs in active smokers compared to non-smokers [71]. In vivo and in vitro studies on nicotine exposure demonstrated the activation of several downstream signaling pathways in fibroblast and airway epithelial cells [80, 81]. In active smokers, bronchial epithelial cells express less the β4 subunit of nAChRs than ex-smokers and never-smokers [82]. Chronic nicotine exposure increases the expression of the α5 subunit of nAChRs in epithelial cells. The α3 subunit is also decreased in airway fibroblast cells of smokers [83]. Besides the epithelial and fibroblast cells, many immune cells also express nicotinic receptors. More specific experiments showed that blockade of muscle-type nAChRs effectively prevents nicotine-mediated calcium internalization through PKC and p38, but not p42/44 signaling. P38 is immediately activated after nicotine treatment. The effect of nicotine on different cells is covered in the following sections in more detail. Furthermore, in line with the direct interaction with nAChRs, nicotine indirectly affects the renin-angiotensin system. Exposure to nicotine increases the expression of ACE2 receptor in airway cells. This upregulation, along with the immune system’s misregulation, is discussed in hypersensitivity pneumonia and coronavirus sections.

## Cellular physiology in response to nicotine

Following inhalation of tobacco smoke, nicotine is absorbed mainly by small airways and alveoli of the lungs, and the residue is distributed throughout the body by the circulatory system and alters the physiological processes of cells that express nAChRs. In addition to lungs, nicotine has profound systemic effects on many organs such as kidneys, heart, liver, and gastrointestinal tract, mediated through damage to epithelial, endothelial, and fibroblast cells [84]. Besides, nicotine can massively affect the function of the immune system [85]. As the first line of defense against pathogens, the innate immune system constitutes chemical and physical barriers (such as skin and other epithelial surfaces) and cellular defenses. Dendritic cells (DCs), monocytes and macrophages, Langerhans cells, neutrophils, natural killer (NK) cells, basophils, eosinophils, and mast cells are the cells of the innate immune system. Having the ability of processing and presentation of antigens to specific types of lymphocytes makes macrophages and DCs as two professional antigen-presenting cells (APCs). Likewise, the adaptive (acquired) immune system provides a specific immune response with an immunological memory against infectious agents. T cells and B cells play essential roles in the adaptive immune defense [86].

**Fig. 3:**
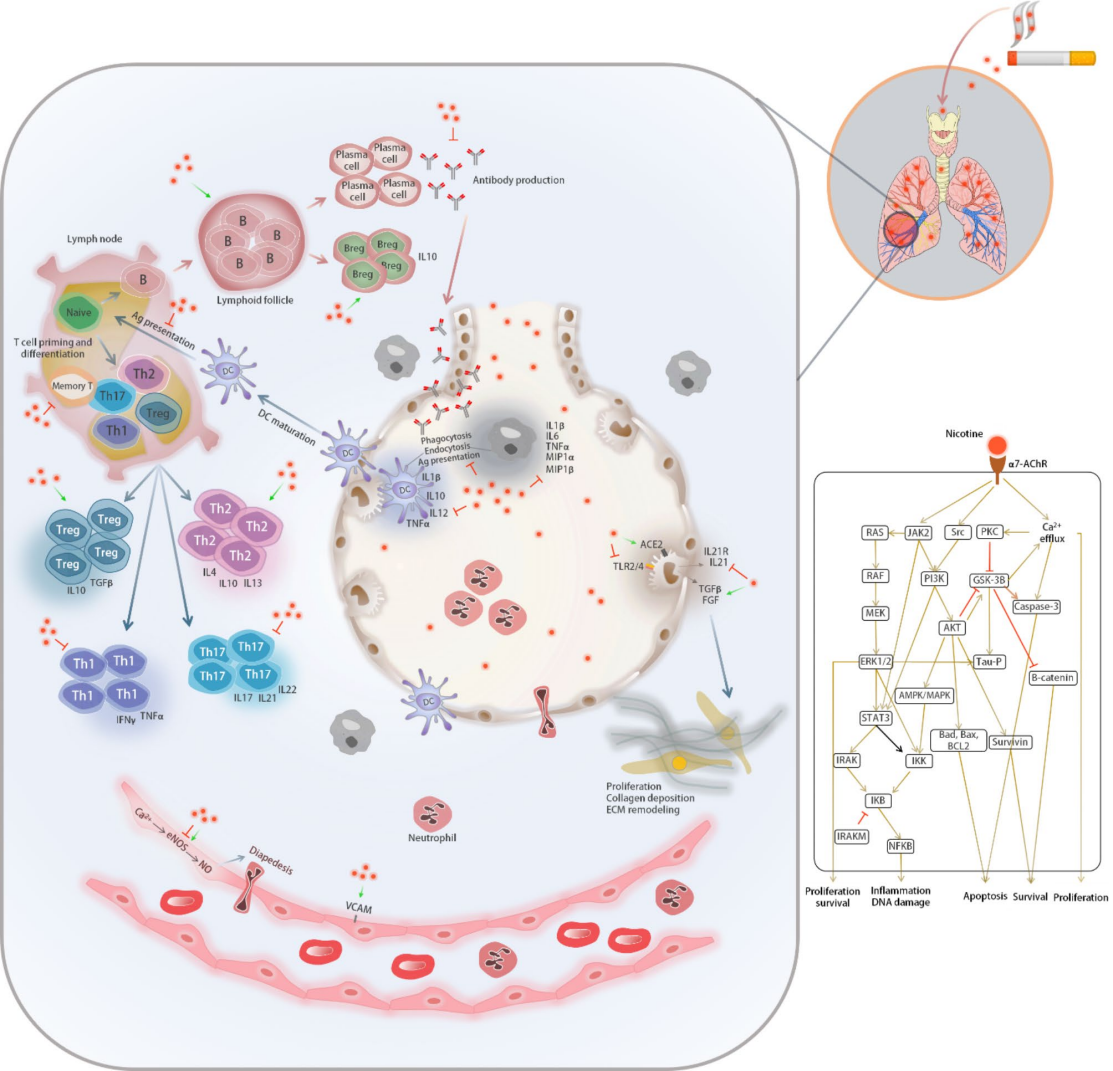
An illustration of the effects of nicotine in suppressing or activation of different signaling pathways. An abstract signaling pathway indicating the effect of nicotine via the nAChRs receptor is also provided

Depending on the cell type and combination of the various subunits of nAChRs, nicotine contributes to releasing growth factors, modification of extracellular matrix, dysregulated growth, and angiogenesis. Herein, nicotine’s effect on its primary targets includes epithelial, endothelial, fibroblast, and immune cells like DCs, macrophages, T, and B cells are covered (Fig. 3).

### Epithelial cells

Epithelial cells form a mechanical barrier that prevents pathogenic substances and secrete mucus and antimicrobial peptides in host defense. Nicotine changes the viability, morphology, and motility of epithelium in various organs, including the respiratory system and oral mucosa [87–89]. Nicotine increases the expression of kidney injury molecule-1, the classic epithelial-mesenchymal transition (EMT) markers, vimentin, fibronectin, and production of α-smooth muscle actin (α-SMA), TGF-β, MCP-1, and ROS in the renal epithelium. Nicotine contributes to renal injury via STAT3, JNK, and AP-1 [90, 91]. In addition to the increased frequency of eosinophils in lamina propria (a thin layer of connective tissue), nicotine enhances the expression of ICAM1 on the submucosal connective tissue of the intestine and trachea [92]. Upon exposure to nicotine, the release of granulocyte-macrophage colony-stimulating factor (GM-CSF) and the activation of Akt are also stimulated in airway epithelial cells (AECs) [93, 94]. In response to chronic nicotine exposure, the upregulation of nAChRs occurs in bronchial epithelial cells (BECs), leading to the activation of PKC and MAPK p38. Similarly, the p42/44 signaling pathway is activated in airway fibroblasts [71]. The presence of nicotine also suppresses inflammatory factors such as MUC5AC in airway epithelial cells [95]. The bronchial epithelial cell showed apoptosis and senescence via ROS mediated autophagy-impairment mediated by nicotine [96]. Same to macrophages derived from human monocytes (MDMs) and type II pneumocytes, nicotine decreases the expression of the TLR-2, TLR-4, and NOD-2 on epithelial cells, which happens without alteration of the viability of the cells. Nicotine also reduces the expression of the surfactant protein (SP)-D in type II pneumocytes. During Mycobacterium tuberculosis infection, nicotine decreases IL6 and CCL5 in epithelial cells (EpCs), whereas in MDMs, there is a decrease in IL6, IL8, IL10, and TNF-α, and CCL2, CXCL9, and CXCL10. The authors pointed that nicotine probably modulates the expression of these molecules by an independent nAChRs pathway [97]. IL22 cytokine is a vital link between immunity and barrier function. By increasing transepithelial resistance and epithelial cell regeneration and repair, IL22 pathway maintenances epithelial cell health and the lung function [98]. Nicotine also downregulates the expression of IL-22Rα1 which is followed by suppression of AECs response to IL22 [99].

### Fibroblasts

As the primary source of the extracellular matrix (ECM), fibroblasts provide the structural framework for tissues and play a critical role in wound healing [100]. Over the past decades, many studies have provided evidence for nicotine involvement in the modulation of fibroblast activation and function in various systems, such as the heart, lung, gingiva, prostate, and joints. However, gene expression changes may not be identical. Also, nicotine interrupts the airway epithelium’s barrier function, leading to the release of inflammatory proteins into the systemic circulation and causes multi-organ fibrosis [101]. TGF-β is described as the key molecule responsible for nicotine-induced fibrosis in various organs [102–104].

Further, nicotine administration results in increased fibronectin (FN1) gene expression in rat hearts [105]. Nicotine also alters the deposition and phagocytosis of collagen in fibroblast cells [106]. In a dose-dependent manner, nicotine raises nerve growth factor (NGF) secretion by lung fibroblasts and stimulated NF-κB nuclear translocation and transcriptional activity [107]. Both fibroblast proliferation via MEK-1/ERK and their collagen type I expression in the lung via α7nAChRs are also induced by nicotine [108]. Also, nicotine tempers fibroblast proliferation via modulation of growth factors and microRNA, particularly by targeting miR-24 [109].

### Endothelial cells

The endothelium is a thin layer of cells between the interior surface of blood vessels and lymphatic vessels. They have various tasks like; permeability, leukocyte trafficking, vascular tone regulation, angiogenesis, and immunity. Hence, the impaired endothelial function leads to inflammatory diseases, remarkably vascular disorders (i.e., atherosclerosis) [110]. Nicotine is one of the main components of cigarettes that can cause endothelial dysfunction. Nicotine exposure gives rise to differential gene expression of endothelial cells, growth of atherosclerotic plaques, and angiogenesis [111, 112]. Human coronary endothelial cells treated with nicotine showed increased expression of ACEI, nitric oxide synthase, and vascular cell adhesion molecule-1 (VCAM1) [113]. Angiotensin II Type I Receptor Antagonism (AT1) was found to diminish the adverse effects of nicotine on cardiac structure and function [105]. nAChRs are also expressed on the upper respiratory endothelial cells, neighboring to ACE-2 receptors [114].

### Dendritic cells

Since DCs facilitate the initiation of cell-mediated adaptive immune responses, they are pivotal cells of the innate immunity system. Dendritic cells are well-known as the most professional APCs with high expression of major histocompatibility complex (MHC) class II molecules (MHC-II), and they can uptake, process, and present antigens to naïve T cells and promote their polarization into different T cell subsets [115]. Dendritic cells can uptake antigens via several mechanisms such as receptor-mediated endocytosis (using mannose receptor, FcγRI, and FcγRII), macropinocytosis, and phagocytosis (via CD36 and αvβ3 or αvβ5 integrins) [116]. Although rare in human blood, DCs are heterogeneous cell populations with different subsets, including plasmacytoid DC (*pDC*) and classical DC or myeloid DC (cDC). The latter can further be divided into two subpopulations of *cDC1* and *cDC2*. The DCs subsets have mouse counterparts as well [117].

Aicher et al. showed that nicotine could enhance HLA-DR expression, costimulatory molecules (CD86 and CD40), and adhesion molecules (LFA-1 and its ligand, CD54) in human DCs. Therefore, nicotine increases the production of IL12 in DCs and stimulates the activation of T cells, which is followed by augmented IL2 production and increased CD40L expression. They proposed that ERK1/2, p38 MAPK, and Akt signaling pathways may mediate the effects of nicotine on DCs [118]. For the first time, Nouri-Shirazi et al. provide evidence of the immunosuppressive effect of nicotine on human DCs. They could demonstrate that nicotine exposure alters the function of monocyte-derived DCs, including **(1) antigen uptake**: no significant change was observed in macropinocytosis, but endocytic and phagocytic properties of immature DCs were reduced. In this context, they observed downregulation of mannose receptor without a change in CD36 expression; **(2) maturation and cytokine production**: DCs matured in response to lipopolysaccharide; however, they produced a lower level of pro-inflammatory cytokines, including IL1β, IL10, IL12, and TNF-α; and **(3) T cell responses**: in the presence of nicotine, DCs showed an anti-proliferative effect on T cells which finally led to the inhibition of Th1 polarization and IFN-γ production. The latter was linked to the downregulation of IL12 as well as CD86 (B7.1) and CD80 (B7.2) in human DCs [119, 120]. Correspondingly, Nouri-Shirazi et al. provided similar evidence for nicotine’s effect on mouse DCs [121]. Later, the association of nicotinic environment and dose-dependent functioning of DCs was described; The pro-apoptotic activity in DCs in high doses of nicotine and anti-apoptotic effect in the lower doses [122]. In a recent study, nicotine-treated DCs showed increased expression of CD40 and CD197 and decreased expression of CD86, MHC-II, and CCP3 (an apoptotic molecule). Interestingly, nicotine did not alter CD80 levels in DCs. While the phagocytosis property of DCs was attenuated in the presence of nicotine, no change was seen in its endocytosis function [123].

### Macrophages

Macrophages, as mononuclear phagocytes, are highly heterogeneous cells that differentiated from monocytes. They have multiple functions, such as phagocytosis, antigen presentation, and the production of different types of cytokines [124]. Based on their role, several distinct subsets of macrophages have been defined: **(1)** classically activated macrophages (*M1*) which mediate antimicrobial defense via secretion of pro-inflammatory cytokines, **(2)** alternatively activated macrophages (*M2*) with anti-inflammatory function, **(3)** regulatory macrophages (*Mregs*) which secrete large amounts of IL10, **(4)** tumor-associated macrophages (*TAMs*) which suppress anti-tumor immunity, and finally **(5)** myeloid-derived suppressor cells (*MDSCs*) as a group of immature cells linked to TAMs [125].

It has been reported that exposure of human macrophages to nicotine increases the intracellular ROS through the activation of Src/PI3K/Akt cascade and NADPH oxidase, which activates AMPK/MAPKs and NF-κB signaling. Flowingly, the activation of these signaling pathways upregulates the expression of IL8, a pro-inflammatory chemokine [126]. While nicotine upregulates the expression of IL8, the expression of IL1β, IL6, TNFα, MIP-1α, MIP-1β, and MCP-1 are downregulated. It also attenuates the phagocytosis of macrophages by affecting phagocytic recognition molecules, SR-A1 and TLR-2. [127]. However, macrophages treated with nicotine exhibited upregulated expression of CD36, possibly via the ROS/PKCδ/PPARγ signaling pathway [128]. The exposure of mice to nicotine also showed a rise in macrophages’ infiltration into the bronchoalveolar lavage fluid [129]. Besides the induction of inflammatory macrophages, nicotine treatments promote the M2 polarization, reduces the secretion of several proinflammatory cytokines in macrophages, and suppresses the alveolar macrophages to present the inhaled antigen to lymphocytes [130, 131].

In human monocytes, activation of α7nAChR inhibits IκB phosphorylation, followed by inhibition of nuclear localization and transcriptional activation of NF-κB [132]. Likewise, in murine macrophages, the activated α7nAChR can mediate Jak2-STAT3 activation and then can induce the phosphorylation of STAT3, which subsequently downregulates pro-inflammatory cytokines (e.g., IL6, IL12, and TNF-α) [133]. Similarly, overexpression of IRAK-M (a negative regulator of TLR-mediated immune responses) in human macrophages through JAK2/STAT3/PI3K not only mediates the anti-inflammatory effect of nicotine via α7nAChRs but also triggers the hyperresponsivity [134]. Altogether, these data suggest that nicotine exerts a dual role in macrophages. In a recent study, it has been shown that the effect of nicotine on infected macrophages is contradictory to uninfected macrophages; **(1) uninfected** macrophages express anti-inflammatory responses, including polarization of CD206^+^ M2 macrophages and increased expression of IL10, whereas **(2) infected** macrophages show inflammatory responses, including differentiation to M1 macrophages and increased expression of iNOS, TNF-α, and IL6. Additionally, they have identified a dose-dependent effect of nicotine on macrophages through α7nAChRs [135].

### T cells

Once developed in the thymus, T cells circulate throughout the body to participate in the cell-mediated adaptive immune response. Overall, there are two specific T cells, including CD4^+^ T cells (helper T cells; Th) and CD8^+^ T cells (cytotoxic T cells; Tc). The CD4^+^ Th subset includes Th1, Th2, Th9, Th17, Th22, Treg, and Tfh categorized according to their surface molecules, transcription factors, and functions. In parallel, there are such subsets for CD8^+^ T cells [136].

Many studies have been performed to show how nicotine alters T cell-mediated immune responses. With a dose-dependent response, the serum level of cotinine, the primary metabolite of nicotine catabolism, was correlated with increased naive (CD45RA^+^) and decreased memory (CD45RO^+^) CD3^+^ CD4^+^ T cells. However, the CD8^+^ T cell population showed no significant difference in passive smokers than controls [137]. Besides α7nAChRs, nicotine appeared to increment Th2 cells via the activation of α4nAChRs induced through the Gprin1/CDC42 signaling pathway. The α4nAChRs-expressing Th lymphocytes were found in the circulation system, spleen, bone marrow, and thymus [138]. Additionally, nicotine-treated mice showed a higher frequency of PD-1^-^ IL7R^+^ CD8^+^ T cells (non-exhausted phenotype associated with loss of tolerance) in bone marrow and spleen. These cells were promoted to produce survivin. In parallel with higher expression and activation of α4/α7nAChRs, the transcription factors of T-bet and Blimp-1 were downregulated in CD8 + T cells [139].

In the context of nicotine’s immunosuppressive properties, a pivotal role has been attributed to the expression and activation of α7 nAChRs on CD4^+^ T cells. Nicotine skewed polarization from Th1 and Th17 cells to Th2 cells. Although both Th1 (IFN-γ and TNF-α) and Th17 (IL17, IL21, and IL22) cytokines were downregulated, the Th2 cytokine (IL4) was upregulated. This effect was further accompanied by reducing T-bet and augmentation of GATA-3 and lower NF-κB-mediated transcription of I-κB and IL-2 [140]. According to a recent study, the co-culture of CD4^+^ T cells and DCs in a nicotinic environment reduced the differentiation to Th1 (IFN-γ), increased the differentiation to Treg (FOXP3) and Th2 (IL6, IL10, and IL13) cells [123]. Nicotine stimulation also reduced the proportion of IL-22-producing PBMCs, particularly CD4^+^ T cells. By targeting IL-22Rα1 expression, the nicotine impaired IL-22/IL-22R signaling axis [99].

### B cells

B cells are well-known for their ability to support the antibody-mediated immune responses along with their immunoregulatory functions. Typically, B cells are characterized as CD19^+^ and B220/CD45R^+^ cells. There are various types of B cell populations, including (1) B1 cells (CD19^+^ and B220^low/-^), which can be detected in most tissues, (2) follicular B cells (*FO* or *B2*), (3) marginal zone B cells (*MZ*), (4) transitional B cells, and (5) regulatory B cells (*Bregs*); Breg-derived IL10 has a pivotal role in tolerance and differentiation of Treg cells [141–143].

Based on a preliminary study, the lack of nicotine-binding sites was shown on B lymphocytes [144]. Later, as the first demonstration, both α4- and α7nAChRs were identified on nicotine treated B lymphocyte-derived cell lines. The authors concluded that long-term exposure with nicotine led to the upregulation of nicotinic receptors coupled with increased cell proliferation and suppressed antibody production. The nAChRs regulated B lymphocyte activation and immune response through CD40 signaling pathway [145, 146]. Skok et al. further confirmed a higher count of B cells (lymphopoiesis) in nicotine-treated mice. Nicotine-treated B cells develop from their early precursor, pre-B cells (B220^+^ IgM^-^ CD43^-^) [147]. They have recently reported that not only CD5^+^ and Foxp3^+^ B lymphocytes are enriched with a high amount of α7nAChR, even it is necessary for the formation, induction, and functioning of regulatory B lymphocytes [148].

Overall, nicotine influences on immune cell responses seem to be profoundly dependent on experimental design, nicotine concentration, and exposure to nicotine. As discussed below, it is plausible that environmental signals, including autoimmune and infectious disease models, also implicate in the fate of nicotine treated cells. More studies are required to assess both inflammatory and anti-inflammatory effects of nicotine on cells of the immune system.

### Neutrophils

Neutrophils are the most abundant type of leukocytes in blood circulation in humans. They are known as a main part of the innate immune system and are the first cells that migrate toward inflamed or infected sites [149]. There are few studies on the effect of nicotine on neutrophils. In the study of Iho et al. the blood level of IL-8 elevated in smokers compared with non-smokers. Increased production of IL-8 was observed in nicotine-stimulated neutrophils in a time- and concentration-dependent manners. Nicotine-induced IL-8 formation is mediated via nAChR, depends on peroxynitrite generation and following NF-κB activation [150]. Also, nicotine stimulates neutrophils to release neutrophil extracellular traps (NETs) in a dose-dependent manner. nAChRs through activation of Akt and PAD4 and without activation of Nox2 produce nicotine-induced NET. These results show that nicotine can be involved in smoking-related diseases [151].

### Mast cells

Mast cells play a key role in inflammation caused by allergic reactions [152]. They express receptors for IgE (FcεRI) which bind to allergens in vivo and induce the release of Th2 cytokines and cysteinyl leukotrienes (cysLTs) [152–156]. Activated mast cells are involved in pathologies of some pulmonary diseases such as COPD, emphysema, asthma, and COVID-19 [27, 157–159].

Several studies investigated the mechanism by which nicotine may affect mast cells. An in vitro study showed nicotine could suppress the delayed phase of activated mast cells via α7/α9/α10 nAChRs and could inhibit the cytosolic phospholipase A2/MAP kinase pathway [160]. Another in vitro study demonstrated cigarette smoke medium (CSM) suppressed *c-kit* and FcεRI expression in bone-marrow-derived mast cells (BMMCs) and inhibited the development of mast cells in a toll-like receptor 4 (TLR4)-independent manner [161]. TLR4 a protein that play the role in the maturation of DC and B cells [162, 163]. Exposure of RBL-2H3, as a mast cell model, to cigarette smoke condensate (CSC) stimulated MCPTs, especially tryptases, secretion. In line with this finding, several in vivo investigations showed there is an adverse correlation between MCPTs levels and airway function, suggesting the contribution of MCPTs in airway remodeling in smokers [164]. Increases in mucosal mast cells (MMCs) were shown in the colonic mucosa from mice with food allergy [165]. nicotine inhibited the activation of MMCs via *a*7 nAChRs and induced the expression of cytokines (Th1 and Th2 types), subsequently ameliorated food allergy mice [166].

## Nicotine in diseases

Smoking inserts a high amount of xenobiotics, which in turn can alkylate DNA, activate specific (anti)inflammatory signals, and oxidative stress in active smokers and also second-hand smokers [167, 168]. This makes smoking a major risk factor for some diseases such as COPD, lung cancer, and diabetes. As a challenging concept, while there is a constant inflammation in smokers, there is protection against particular inflammatory diseases, like obesity and uncertain colitis [169]. Whereas ulcerative colitis is reversed after smoking and is worsened after smoking cessation, Crohn’s disease is exacerbated following smoking [170–172]. Consequently, smoking can be a double-sided sword. As we have mentioned earlier, the effects of cigarette smoke, although similar in some effects, can be different from pure nicotine [173–176]. The receptor of α7nAChR is one of the primary candidates to role in this process. Generally, activation of the acetylcholine-α7nAChR signaling axis by acetylcholine or nicotine inhibits NF-κB, the critical transcription factor in the activation of inflammation in different immune cells [174, 175, 177].

**Fig. 4:**
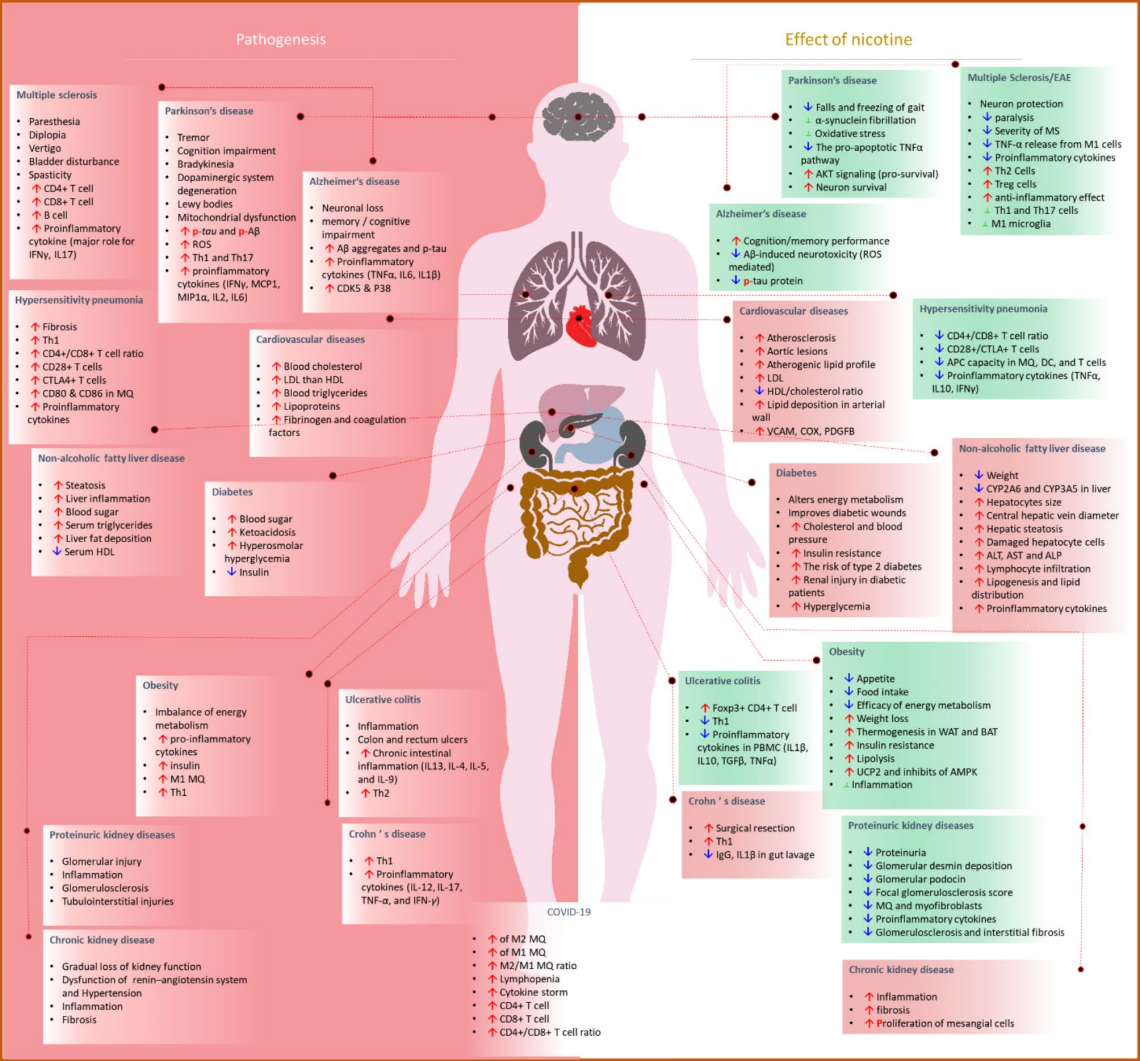
Nicotine shows a dual effect in different diseases. A graphical abstract of major pathogenesis of 13 diseases in several organs (left) and the effect of nicotine (right), in amelioration (light red) or worsening (light green), of the diseases are shown

In the following sections, we discuss nicotine’s influence and, to some extent, smoking on various model inflammatory/autoimmune diseases affecting different organs, including respiratory, nervous, metabolic, digestive, cardiovascular, and urogenital systems. Mainly, we will review the effects of nicotine on coronaviruses (Fig. 4).

### Smoking and Post-COVID − 19 symptoms

According to a WHO report based on the monitoring of patients with COVID-19 after recovery in 2022, these people, particularly those with co-morbidities, displayed many symptoms for months after recovery [178]. Post-acute COVID-19 syndrome, also known as ‘post-COVID syndrome,‘ ‘long COVID,‘ ‘persistent long COVID,‘ or ‘post-acute COVID sequelae (PACS),‘ is a pathological disease characterized by persistent physical, medical, and cognitive sequelae after acute COVID-19 [179]. Post-acute COVID-19 syndrome, like COVID-19, can impact various systems and organs, including the respiratory, cardiovascular, neurological, gastrointestinal, and musculoskeletal systems [180]. There is significant evidence that post-acute COVID-19 syndrome can impact the whole COVID-19 patient population, and that around 20% and 10% of SARS-CoV-2 positive patients have symptoms that last at least 5 or 12 weeks, respectively [181].

The number of research on the influence of smoking on Post-COVID-19 symptoms is limited, and we will discuss the scant information that exists in this area in this section. According to the results of a 2022 study done in France, smoking can contribute to a rise in Post-Covid-19 symptoms such as cutaneous disorders, tachycardia and/or hypertension. In fact, this study discovered a link between smoking in women and the occurrence of these symptoms following recovery [178]. Another study found that the majority of COVID-19 recovered patients have varying degrees of functional impairments ranging from insignificant to severe based on Post-COVID-19 Functional Status (PCFS). Age, gender, periodic influenza vaccination, smoking, length since symptoms began, requirement for oxygen or ICU admission, and lastly the existence of concomitant comorbidities all had an impact on these limits [182]. Based on the findings of a 2022 study, smoking and vaping are not only risk factors for a more severe clinical form and slower progression of COVID-19, but they are also risk factors for the development of lengthy and persistent post-COVID-19 symptoms [183]. Finally, it is crucial to note that all of the above cases are associated to smoking and post-COVID-19 symptoms, and these cases cannot be attributed to nicotine alone; additional research in this area is required.

### Nicotine in Diseases of Respiratory System

#### Hypersensitivity pneumonia

Hypersensitivity pneumonia (HP), also known as extrinsic allergic alveolitis, is an inflammatory lung disease associated with lymphocytes’ accumulation. Numerous predisposing factors such as genetics and environmental stimuli and lifestyle are involved in developing the disease. Chemical, bacterial, fungal, proteogenic factors, and animal antigens are among HP’s critical risk factors [184].

HP is classified into acute, subacute, and chronic categories and Th1 polarization plays a significant role in developing acute/subacute HP and stimulation of reversible granuloma formation. Afterward, this response continues to Th2 response in chronic HP and promotes inflammatory responses and fibrosis [185]. Increased CD4 + T cells and increased CD4+/CD8 + ratio along with decreased CD8 + are observed in chronic HP. Fibrotic HP is very similar to idiopathic pulmonary fibrosis (IPF) and is probably responsible for recruiting fibrocytes and the activation of fibroblasts, and therefore, the uncontrolled deposition of ECM-related proteins [186]. The CD80 and CD86 molecules are expressed on the APC cells (macrophages and dendritic cells, monocytes) and bind to CD28 and CTL4 molecules on T cells. The CD28 marker is expressed on resting T cells, and its stimulation causes the activation and proliferation of T cells. The CTLA4 marker is also expressed on T cells after stimulation [187]. In HP, changes in T cells (CD28 + and CTLA4 + T cells) and macrophages surface markers are reported. These molecules play essential roles in the accumulation and proliferation of lymphocytes and the expression of TNF-α, IL10, and INF-γ in HP patients [187]. While Alveolar macrophages naturally show a limited expression of B7 and have little capacity to act as APCs, macrophages show higher APC capacity in HP. Interestingly, viruses play a pivotal role in HP pathogenesis acting via B7 in alveolar macrophages [188]. So, inhibition of the costimulatory molecules on macrophage and T cells prevents the development of HP.

Although smokers with chronic HP develop more recurrent periods and have lower survival rates, it has been shown that HP is less common in smokers than non-smokers. The mechanism by which smokers are protected against HP is unclear [189, 190]. Animal studies indicate the anti-inflammatory effects of short-term nicotine exposure, while its long-term effects can cause inflammation and fibrosis [190]. Nicotine also suppresses monocytes and macrophages’ APC activity to display the inhaled antigen to lymphocytes, leading to reduced immune reaction toward HP [189].

### Nicotine in Diseases of Nervous System

While neurodegenerative diseases appeared to be more prevalent in smokers than non-smokers, epidemiological studies during the early 1960s provide the first evidence of more smoking is used, less incidence of Parkinson’s disease is observed [191, 192]. Since then, several studies have proven the critical role of nAChRs activation in modulating neuro-immune pathways, and nicotine has been investigated as an effective therapeutic in neurodegeneration and neuroinflammatory diseases [193, 194]. Although nAChRs are widely distributed in different nervous system regions, nicotine neuroprotection in the brain and spinal cord is mainly mediated via α7nAChRs and α4β2 [195]. Below we will review the impact of nicotine in Alzheimer’s disease (AD), Parkinson’s disease (PD), and multiple sclerosis (MS), which are among the major cause of neurological impairment in middle and late life [196].

#### Alzheimer’s disease

Alzheimer’s disease is the most common progressive neurodegenerative disease, which accounts for 60 to 80% of the world’s 47 million dementia cases [197]. Patients typically display with neuronal loss, memory, and cognitive impairment at the disease onset. According to the degree of cognitive impairment, there are different types of AD [198]. Aggregation of misfolded proteins is a key feature of AD. In the case of amyloid b-protein (Aβ) aggregates and tau hyper-phosphorylation (p-tau) induced pathogenesis, neuronal damage occurred through proinflammatory cytokines (IL1β, IL6, and TNF-α) or increased activity of CDK5 and P38 [199]. Among environmental components, smoking is a significant risk factor for AD, and smoking-related oxidative stress influences the Aβ and tau aggregation [200]. However, nicotine extracted from tobacco may exert neuroprotective effects in AD [201].

Transdermal and intravenous nicotine treatment has been shown to improve cognitive dysfunction in AD patients [202, 203]. In an ongoing Phase II clinical trial (MIND), a nicotine skin patch is examined for memory performance in older adults (ClinicalTrials.gov identifier NCT02720445). Nicotine reduces the Aβ-induced neurotoxicity relevant to oxidative stress through regulating metal homeostasis [204]. Recently, it has been shown that even at a 10:1 ratio, nicotine does not have a significant effect on Aβ and its aggregation pathway and may affect Aβ amyloid burden by affecting Aβ production, degradation, or/and localization [205]. Furthermore, “*increased expression of BAG2 shifts the effects of nicotine toward a reduction in levels of p-tau protein, possibly as a consequence of BAG2 inhibition of ERK1/2 via association with Hsp90, which is required for ERK1/2 activity, and/or via BAG2-mediated degradation of p-tau as a consequence of BAG2 phosphorylation by p38/MAPKAPK2*” [206].

#### Parkinson’s disease

As the second most common neurodegenerative disease following AD, Parkinson’s disease (PD) affects older individuals. Parkinson’s disease has both slow and rapid progressive pattern. Tremor, cognitive deficits, bradykinesia, and anxiety are among PD’s main symptoms [207]. Unfortunately, the disease follows a fast increasing trend over the past generations [208]. It has been proposed that genetic and environmental interactions followed by the reactive oxygen species (ROS) production, mitochondrial dysfunction, and formation of Lewy bodies may result in the dopaminergic system degeneration observed in PD. The Lewy bodies mainly include misfolded proteins like alpha-synuclein (SNCA), phosphorylated tau, and Aβ protein [209]. In addition to altered T cells subpopulation (significantly more Th1 and Th17 cells), increased proinflammatory cytokines such as IFN-γ, IL2, IL6, TNF-α, MCP-1, and MIP-1α also point to the role of an autoimmune reaction in PD pathogenesis [210, 211].

Regarding PD, cumulative evidence has provided clues about nicotine’s neuroprotective role, which suggests the implication of nicotine and some of its derivatives for PD therapy. Indeed, some epidemiological findings show a decreased risk of PD in smokers [212, 213]. Previously, nicotine patches and gums have been shown beneficial effects in PD patients [214, 215]. However, the use of nicotine patches has shown conflicting results [216]. Recently, PD patients prescribed by an oral formulation of nicotine (NC001) (ClinicalTrials.gov identifier NCT00957918) have shown improvement in falls and freezing of gait (FOG) [217]. An early clinical trial (ClinicalTrials.gov identifier NCT03865121) also proposed nasal administration as the optimal nicotinic therapy in PD patients. Various mechanisms of action have been attributed to the protection of nicotine against PD, mediated through both nicotinic, mainly α7nAChRs, and non-nicotinic receptors [218, 219]. Nicotine treatment significantly suppresses proteins α-synuclein fibrillation [220]. Enhancement of fibroblast growth factor-2 (FGF-2) and brain-derived neurotrophic factor is another neuroprotective mechanism of nicotine in PD [221]. According to recent research, nicotine has been assumed to protect from PD by suppressing manganese and iron-induced oxidative stress [222]. Interestingly, by inhibiting SIRT6, nicotine downregulates the pro-apoptotic TNFα pathway and activates the pro-survival AKT signaling, which ultimately promotes neuron survival in PD [223].

#### Multiple sclerosis

Multiple sclerosis is the most common autoimmune-mediated demyelination and disabling disease of the CNS in young adults between 20 and 40 years. More than 2.5 million people worldwide are diagnosed with MS, and it follows an increasing pattern of prevalence [224]. MS is characterized by moderate-to-severe spasticity, paresthesia, dysesthesias, diplopia, ataxia, vertigo, and bladder disturbances [225]. Activation and differentiation of T cell subsets (IFN-γ and IL17 producing CD4 + and CD8 + T cells) and antibody production of B cells have a pivotal role in disease initiation and progression [226]. Through inducing an anti-inflammatory effect, nicotine has been explored to attenuate MS and experimental autoimmune encephalomyelitis (EAE), a mouse model for MS.

There is spars literature about the effect of nicotine on MS. Overall, the studies show an inverse correlation between snuff and the risk of MS [227, 228]. Under the EAE condition, nicotine has been shown to suppress the production of Th1 and Th17 cytokines and increase anti-inflammatory Th2 and immunosuppressive Treg cell responses in the CNS [140, 229]. Nicotine treated mice also showed delayed onset and a significant reduction in the severity of EAE coupled with less inflammation in histopathologic evaluation [230]. Moreover, nicotine suppresses M1 microglia cell differentiation and decreases TNF-α release from these cells, impairs Th1 and th17 cells differentiation which consequently resulted to the protection of neurons [231]. The ability of nicotine to lessen the infiltration of CCR2^+^ Ly6C^high^ monocyte and neutrophil into the CNS of nicotine-treated EAE mice is mediated by both α7- and α9nAChRs. Notably, nicotine reduced the mRNA transcript levels of CCL2 and CXCL2 chemokines involved in the chemotaxis of pro-inflammatory monocytes and neutrophils, respectively, in the brain of EAE mice [232]. Recently, it has been explored that the therapeutic effect of nicotine in CNS inflammation requires the presence of B1a lymphocytes. Moreover, binding of nicotine to α7nAChR reduces the secretion of proinflammatory cytokines (TNF-α, IL-1β, IL6, and IL18) from LPS-stimulated human monocytic cell line and diminishes paralysis in EAE [233].

### Nicotine in Diseases of Metabolic System

#### Obesity

Obesity is the extra or abnormal accumulation of fat in the body and is an essential health-threatening factor [234]. Fatness is usually calculated as body mass index (BMI; body weight in kg divided by the square of the height in meters). Accordingly, the worldwide prevalence of overweight (BMI ≥ 25 kg/m^2^), obese (BMI ≥ 30 kg/m^2^), and severely obese individuals (BMI ≥ 40 kg/m^2^) are increasing [234]. Notably, obesity is associated with coronary heart disease, end-stage renal diseases, hypertension, type 2 diabetes mellitus (T2DM), osteoarthritis, and fatty liver disease [235].

Nicotine plays a dual role in obesity as a low-grade inflammatory disease. Maternal nicotine consumption not only dysregulates energy metabolism, including hormonal imbalance (leptin, insulin), even it increases the risk of metabolic syndromes such as obesity and hyperleptinemia and larger adipocytes in the offspring during adulthood [236, 237]. In obese patients, nicotine’s positive effect on energy metabolism, including glucose and insulin, is associated with the suppression of inflammatory signaling pathways [238]. There are also reports indicating nicotine involvement in weight loss via induction of thermogenesis in brown adipose tissue and decreasing food intake [239]. Seemingly, nicotine suppresses weight gain and obesity independent from food uptake [240]. Smokers and COPD patients, who suffer from weight loss, are less prone to obesity, and smoking cessation is associated with weight gain in active smokers [241, 242]. Nicotine’s negative energy regulation is mediated via AMP-activated protein kinase (AMPK) [243, 244], that plays a central role in the regulation of feeding and energy metabolism. Nicotine increases energy metabolism and decreases the efficacy of energy conversion [245]. As well, nicotine downregulates the expression of CCL2 and F4/80 in adipose tissue, which diminishes the infiltration of M1 macrophages [246].

Additionally, nicotine induces insulin resistance via the MKP1-P38MAPK-cJun-IRS1 signaling axis and mTOR activation in skeletal muscle cells, which intensifies lipolysis and weight loss [247]. Nicotine can regulate appetite and food intake genes. For example, nicotine induces uncoupling protein1 (UCP1), a protein involved in thermogenesis in BAT, in white and brown adipose tissues [248, 249]. Also, nicotine seems to up-regulate the neuropeptide Y (NPY) and orexins, molecules for feeding, while down-regulate their receptors [249].

#### Diabetes

Diabetes affects many organs, including the heart, kidney, eye, and nerve system, and induces foot pain and ulcers. Smoking increases the risk of T2DM by about 30–40% (women > men), and after secession, weight-gain is more in women [250]. Smoking intensifies the complications of diabetes, and the combination of smoking and diabetes increases the mortality and mortality rates. Diabetic smokers also have a higher level of cholesterol level and blood pressure. Besides, diabetic smokers control their blood sugar with larger insulin doses and cause resistance to insulin. Animal studies demonstrated adiposity and metabolic changes in fetal and neonates after nicotine exposure [251]. Remarkably, smoke during pregnancy has been shown to increase the risk of T2DM in the offspring. Nicotine also intensifies the renal injury in diabetic mice [252]. Moreover, nicotine uptake increases the blood sugar level due to the alteration of energy metabolism [253]. This regulation is mediated by stimulation of nAChRs in the hippocampus and inactivation of AMPK, and activation of TCF7L26, a diabetes-associated transcription factor. TCF7L2 activates neuronal signaling to the pancreas to release glucagon and less insulin and induction of hyperglycemia (blood glucose level) [254]. Significantly, hyperglycemia inhibits nAChR activity via a negative feedback loop involved in nicotine dependence. Despite all the above, nicotine also has promising effects on diabetic wound healing and angiogenesis in genetically diabetic mice. The distribution of nAChRs in endothelial cells of the mice was stimulated with nicotine and promoted angiogenesis. This function of nicotine-nAChR-angiogenesis was similar to the basic fibroblast growth factor (bFGF) [255].

### Nicotine in Diseases of Digestive System

#### IBD

Inflammatory bowel disease (IBD) is a group of chronic complex inflammatory conditions of the gastrointestinal tract, which is identified as dysfunction and disruption of gastrointestinal epithelial barrier function and increased permeability of the basal membrane. IBD is a life-threatening disease with diverse symptoms ranging from mild to severe, including persistent diarrhea, abdominal pain, rectal bleeding/bloody stools, fatigue, reduced appetite, and weight loss. Ulcerative colitis (UC) and Crohn’s Disease (CD) are two main examples of IBD. Ulcerative Colitis (Th2-mediated) usually occurs in the large intestine and the rectum in which the inflamed areas spread continuously from the rectum toward the colon. However, CD (Th1-mediated) can spread in any area, from mouth to the anus, as patchy inflammatory areas within the healthy tissue [256]. Smoking has different effects on UC and CD; ameliorates UC’s severity but exacerbates CD [257]. While passive smoking is associated with an increased risk of intestinal surgeries in patients with CD [258], UC patients with a smoking history show improvements in their gut function after smoking, and tobacco cessation worsens their gut activity [170, 171, 259]. Remarkably, the mucosal epithelium of UC patients expresses less α3 subunit of nAChRs than healthy controls, possibly because of higher cell renewal [260].

Furthermore, surgical resection is more common in smokers with CD, and their gut lavage fluid contains less IgG, IL1b. This is a contradictory result since it has been demonstrating that smoking worsens CD and nicotine stimulates more inflammatory responses in CD patients [261]. Then, Bergeron, V. et al. demonstrated that there is a dysfunction in mononuclear cells from smokers with CD, and they produce a lower level of chemokines and cytokines in comparison to the non-smokers with CD and smokers with UC. There are also defective anti-oxidative and anti-inflammatory responses in cells from CD patients. Therefore, there is a different antioxidant potential in cells from CD and UC against smoke [262]. A transcriptomics study from colonoscopy samples of CD patients (smokers vs. non-smokers) showed differential upregulation of RNF138, MT2A, and STEAP3 genes in smokers [263].

Besides smoking, transdermal nicotine delivery in UC patients has shown protective effects and improves the symptoms [173, 264]. Some studies in IBD diseases have proposed the immunomodulatory function of nicotine on Th1/Th2 toward Th1, which is perhaps mediated via mir-124-IL6R signaling. The amount of miR-124 expression at the time of nicotine treatment switches the balance toward Th1 or Th2. Nicotine, along with higher miR-124 expression, stimulates Th1 polarization, and lower miR124 expression stimulates Th2 polarization. MiR-124 is downregulated in Crohn’s disease, in which nicotine treatment aggravates the condition toward intensified Th2 responses, whereas miR-124 is upregulated in UC, and nicotine treatment ameliorates the severity of the disease toward Th1 response [6]. Nicotine treatment of LPS-treated PBMC from both UC and CD patients downregulated production of IL1β, IL10, TGF-β, and TNFα [265]. As well, nicotine treated non-adherent mononuclear cells of CD patients, and in vivo nicotine patch showed downregulation of IL10 (Th2 inhibitory function) but did not affect IL2 and TNFα (Th1 responses) [266]. Also, the treatment of DC separated from CD and UC with cigarette smoke extract (CSE) upregulated MHC-II and costimulatory molecules and downregulated the expression of CXCL10 and CCL3 in UC than CD. Whereas CSE in CD increased Th1 polarization, it increased Foxp3 + CD4 + T cells and decreased the Th1 subset in UC samples [267].

#### Non-alcoholic fatty liver disease

Non-alcoholic fatty liver disease (NAFLD) is the accumulation of fatty acid in non-alcoholic individuals and is mostly observed in obese and extremely obese people. Diabetes, overweight, and metabolic syndrome are other risk factors for NAFLD. The disease has two forms, including fatty liver (NAFL) and steatohepatitis (NASH) [268]. Nicotine is genotoxic and can play oncogenic and carcinogenic effects in the liver. By inducing direct and indirect damages in liver cells, nicotine is recognized as a risk factor for several liver diseases such as obesity-induced hepatic steatosis and NAFLD; it can exaggerate them [269].

The adverse effects of nicotine in the hepatic system include the upregulation of inflammatory cytokines, the development of secondary polycythemia, and blockage of proliferation and activation of apoptosis in lymphocytes. An animal study by Willis et al. showed a significant reduction in liver weight and enlargement of hepatocyte diameter and central hepatic vein due to nicotine consumption [270]. Biochemically, rats exposed to nicotine (2.5 mL/kg) showed a significant increase of alanine aminotransferase (ALT), aspartate aminotransferase (AST), and alkaline phosphatase (ALP) in serum due to hepatocyte cell death/damage. Histologically, nicotine increased the inflammation and lymphocyte infiltration in the liver [66]. If combined with a high-fat diet (HFD), nicotine triggers and worsens hepatic steatosis. Besides, hepatic steatosis is associated with higher hepatocellular apoptosis, inactivation of AMPA, activation of acetyl-coenzyme A-carboxylase (ACC), and increases oxidative stress [271]. In HFD, nicotine elevates lipogenesis, lipid mobilization, and distribution and diminishes HFD-induced adiposity, contributing to hepatic steatosis [247, 271]. However, nicotine alone does not trigger hepatic steatosis [271]. Besides toxic effects, nicotine has immunomodulatory effects in the liver [272]. It downregulates and decreases the activity of CYP2A6 in the liver cells of monkeys [273, 274]. Additionally, CYP2A and CYP3A5 are downregulated in the airway of smokers [272, 275].

### Nicotine in Diseases of Cardiovascular System

Cardiovascular diseases (CVDs) are the first cause of death worldwide. As a coronary artery disease, atherosclerosis is a major cause of heart attack, stroke, and peripheral arteries. Generally, chronic nicotine exposure enhances atherosclerosis in animal and human studies. After nicotine administration, impaired and diminished clearance of LDL and accelerated lipid transfer from HDL increases their deposition in the arterial wall, which result in the atherogenic lipid profile (increased LDL, decreased HDL/total cholesterol ratio) [276, 277]. Nicotine also raises heart rate and blood pressure, and platelet aggregation. The latter contributes to plaque growth and thrombosis [278]. Also, nicotine significantly induces vascular smooth muscle cells’ proliferation via the upregulation of bFGF mitogen and several MMPs, which leads to an augmentation of lesions and development of intimal hyperplasia, atherosclerosis, and aneurysm. Chronic nicotine consumption profoundly activates macrophages via nAChRs to express pro-inflammatory cytokines, aggravating atherosclerosis and aortic lesions [279]. Distribution of α7nAChRs on endothelial cells pointedly indicates nicotine-induced angiogenesis in the endothelium [278]. Nicotine induces proliferation, migration, and tube formation in endothelial cells. There is a close association between neovascularization and atherosclerotic plaque progression, which directly increased by nicotine [280]. However, chronic exposure of endothelial cells to nicotine impairs angiogenesis and decreased cell migration and tabular structure formation [281, 282]. In addition, nicotine stimulates the migration of vascular smooth muscle cell and structural changes in vascular endothelium via p38 and p44/42 signaling axis [283]. Treatment with nicotine also activates NF-κB signaling and increases proliferation of endothelial and smooth muscle cells [284].

Additionally, nicotine exposed mice show upregulated expression of VCAM-1, COX-2, platelet-derived growth factor β (PDGFβ), and molecules involved in downstream of NF-κB signaling pathway in aorta lesions [279]. The upregulation of PDGF promotes a phenotypic switching from vascular smooth muscle cells into myofibroblasts and osteoblast-like cells that secrete and modify the endothelium and cardia ECM by collagen and osteopontin [285]. Inhibition of α1nAChR reduced myofibroblasts in the aortic wall by 80% resulting in attenuated calcification and reduced immune cells in the lesions [286]. Treatment of endothelial cells by nicotine induces calcium internalization and accumulation via α7nAChR activation and induction of angiogenesis [287]. Furthermore, via the upregulation of VEGF and FGF, nicotine activates other angiogenic signaling pathways in endothelial cells [288–290]. Therefore, nicotine induces the proliferation of smooth muscle cells, endothelial cells, and accumulation of macrophages in plaques and lesions, promoting atherosclerosis and vessel hardening via calcification.

### Nicotine in Diseases of Urogenital System

Kidneys are essential organ for filtering waste and toxic materials out of the blood. Diseases related to impaired kidney function is increasing with global prevalence of 9·1% (2019) [291]. Nicotine mainly affects kidneys via α7-nAChR signaling and worsens renal injury in chronic kidney disease (CKD), acute nephritis, and subtotal nephrectomy [292]. Different subtypes of nAChRs are distributed in renal cells, especially mesangial cells. Nicotine stimulates differentiation and specialization of cells in kidneys, the proliferation of mesangial cells, and fibronectin production, mediated through PKC activation, ERK1/2 phosphorylation, and NADPH oxidase and ROS generation [252, 292–294]. Also, COX-2 and prostaglandin generation are involved in nicotine-mediated renal injury [295]. Besides, nicotine mediates inflammation and fibrosis by TGF-β production (by activation of STAT3) and upregulation of vimentin, α-SMA, and fibronectin [296].

Several studies have also reported anti-inflammatory responses and attenuation of proteinuric renal injury due to nicotine treatment [297, 298]. Nicotine has renoprotective effects in kidney diseases. Oral nicotine delivery in rats with proteinuria-induced renal inflammation showed dose-dependent reduces proteinuria, glomerular desmin deposition, decreased glomerular podocin, decreased focal glomerulosclerosis (FGS) score, and reduced infiltration of macrophages and myofibroblasts. Renal inflammatory cytokines were also downregulated in monocytes of nicotine treated rats [299]. Furthermore, long-term nicotine delivery has been shown to slow-down proteinuria by reducing glomerulosclerosis and interstitial fibrosis [297, 300–302].

### Impact of nicotine on Coronaviruses

SARS, MERS, and CODIV-19 show similar aggressive pathology. They extensively affect multiple tissues and organs accompanied by uncontrolled cytokine storm and delayed interferon-gamma response, multi-organ fibrosis, impaired tissue remodeling, and organ failure [303, 304]. As mentioned in the former section, nAChRs are expressed in different lung cells, including epithelial cells, fibroblast cells, and RAS components [16]. The effect of nicotine in the renin-angiotensin system is also well studied. Overall, tobacco smoke and nicotine play complicated dynamic functions against coronavirus infections, which are mediated through different mechanisms such as overexpression of ACE2 and inhibition/dysregulation of inflammatory processes [305, 306].

Limited data are indicating the involvement of smoking at an increased risk of MERS infection. Infected smokers during the MERS-CoV epidemic of 2003 showed more mortality rate than infected non-smokers [307, 308]. Alraddadi et al. investigated the smoking history in a population of MERS infected cases and asserted that active smokers are more likely among patients than controls [309]. After this report, Seys et al. reported the upregulation (mRNA and protein level) of dipeptidyl peptidase-4 (DPP4/CD26), the receptor involved in MERS infection lung tissue of smokers and COPD patients. DPP4 is mostly expressed in various alveolar cells, including epithelial, endothelial, macrophages, and other immune cells in the submucosal region of airway epithelium and lymphoid aggregates, but not in bronchial epithelial cells [310]. Also, binding of the spike protein of SARS–CoV-2 to DPP4 is a critical factor for infection [311]. Interestingly, a positive correlation between ACE2 and DPP4 is reported recently in several normal tissues [312]. DPP4 is a natural surface marker for T cells and is involved in the immune regulation of several biological processes. DPP4, the same as ACE2, is much expressed in lower airways, lung parenchyma, interstitium, and pleural mesothelia, which are the most infected areas in infected patients [313–315]. Like DPP4, the upregulated expression of ACE2 has been shown in smokers and COPD patients [316–318]. However, it is downregulated after infection, but ACE remains unchanged, which results in severe acute respiratory failure [319–321]. Imai et al. demonstrated that loss of ACE2 expression in *ace2* knockout mice causes very severe ARDS complications, including increased immune cell infiltration, lung edema, and vascular permeability [319]. Therefore, SARS infection downregulates the expression of ACE2 as a post-infection regulatory mechanism and ARDS [321]. Therefore, the upregulation of ACE2 via nicotine may play a protective role after SARS infections. We could not find any research regarding the direct effect of nicotine in MERS and SARS infections.

Nicotine has a dual effect on patients with COVID-19. On the one hand, nicotine has anti-inflammatory properties, and on the other hand, it would allow more viral entry. Since cytokine storm is the hallmark of SARS-CoV-2 infections, nicotine could diminish it through alpha7-contained nAChRs [322]. Furthermore, agonists of alpha7-nAChRs, nicotine or GTS-21, reduce cytokine storm mediators such as high mobility group box-1 (HMGB1) [323]. HMGB1 is a nuclear protein that is released into the extracellular spaces, including the airways and the blood circulation to promote inflammation [323]. High levels of HMGB1 have been shown in acute lung injury caused by infectious and severe COVID-19 patients, therefore, it could be a therapeutic target for patients with severe COVID-19 [324]. There are controversial reports regarding viral entry by smoking and nicotine use. Some studies showed that ACE2 increased by using nicotine and/or smoking inhabits [325–328]. While Caruso et al. demonstrated that the protein expression of ACE2 diminished in bronchial epithelial cells after smoking exposure [329]. another study reported ACE2 mRNA levels in bronchial epithelial cells from current smokers were similar to never smokers [330]. As mentioned above, in addition to receptors, proteases are needed for virus entrance into host cells. The effect of nicotine/smoking on these proteases is different. The cellular furin levels were reduced by smoking [331], whilst TMPRSS4 and TMPRSS2 increased and unchanged in bronchial epithelial cells from current smokers in comparison with never smokers [330].

Additionally, data mining and *in silico* studies for evaluating the effects of smoking/nicotine on COVID-19 patients were performed. For instance, to determine the relationship between vaping/smoking and the expression of inflammasomes and inflammatory cytokines, Lee et al. used transcriptome datasets (GSE138326 and GSE112073). They found upregulated pro-inflammatory markers and inflammasome genes in smoking and nicotine use as well as e-cigarettes containing flavor. The upregulation of CCL5 and CCR1 in cigarette smoking and CCL5 and CCR1 in vaping e-cigarettes containing flavor/nicotine have been reported. Also, the upregulation inflammasome-related genes (CXCL1, CXCL2, NOD2, and ASC) in smoking and vaping have been observed, revealing the negative effect of vaping/smoking on the inflammation and susceptibility to SARS-CoV-2 infection [328]. Furthermore, *in silico* studies showed interaction of α7 nAChRs with RBDs of SARS-CoV-2 spike glycoproteins, indicating dysregulation of the nicotinic cholinergic system and leading to cytokine storm in COVID-19 patients [332, 333]. Mohammadi et al. demonstrated the interaction between ACE2 human receptor and nicotine by molecular dynamic simulations. They also revealed the combination of favipiravir (antiviral therapeutic for SARS-CoV-2) with nicotine could successfully block 6LZG, a main active site of ACE2-S protein, suggesting the potential influence of this combination for blocking ACE2 versus SARS-CoV-2 [334].

Several systematic reviews and meta-analyses evaluated the association between smoking and COVID-19 hospitalization. Some of these studies showed low smoking prevalence among hospitalized patients with COVID-19, while others demonstrated the inverse trend [335–346]. The latest meta-analysis that included 109 studies with 517,020 patients showed that smoking was related to COVID-19 severity. Also smoking elevated the risk of ICU admission and death in patients with COVID-19, but was not relevant to mechanical ventilation. This study reported former smokers had a risk of progressing COVID-19 severity compared with current smokers. Current smokers were significantly associated with the severity of COVID-19 compared with non-smokers [344]. According to this meta-analysis that has been shown a positive correlation between smoking and COVID-19 progression, it is not clear whether nicotine would prevent negative outcomes among hospitalized patients with COVID-19.

Unfortunately, we were unable to find a publication that looked into the influence of nicotine dosage, age, and COVID disease. However, the findings of other research demonstrate that increasing age is a risk factor for mortality caused by COVID. COPD patients, smokers, quitters, and persons, who have never smoked, on the other hand, have various reactions to this virus [347].

To the best of our knowledge, there is no clinical trial to demonstrate the effects of smoking/nicotine on outcomes in COVID-19 patients. To clarify the effect of nicotine well-designed clinical trials are required for the assessment of its therapeutic agents.

## Conclusion

SARS-CoV-2 infects and affects several body organs, including respiratory, digestive, nervous, cardiovascular, and urogenital systems. The early epidemiological reports indicated the lower incidence of smokers among SARS-CoV-2 infected and hospitalized patients. Then, several letters were published criticizing the higher risk of smoking for the infection. Currently, there is a discrepancy between the published multi-national epidemiological data and current basic data (higher expression of ACE2 in smokers and COPD patients). Of note, the latest meta-analysis that included 109 studies reported that smoking was associated to COVID-19 severity.

As shown in hyper-pneumonia sensitivity, generally similar to SARS-CoV-2 infection, the APC capacity and functioning are reduced in the airways, which attenuates the marshal of adaptive immunity and stimulation of uncontrolled cytokine storm and fibrotic responses. Besides, reports on SARS infections indicate the after-infection downregulation of ACE2 is followed by more severe pathogenesis that produces pro-inflammatory and pro-thrombotic cytokines.

In a brief conclusion, nicotine shows dual effects based on dose-dependent properties. Moreover, in some diseases, although nicotine has a worsening effect (e.g., diabetes) in the pathogenesis of the disease, but has a healing role in the treatment of some other diseases (e.g., multiple sclerosis). Besides, nicotine revealed a dual effect on the severity of COVID-19. Several publications are indicating the overexpression of ACE2 in smokers that cause further viral entry. We conclude that the overexpression of ACE2 in smokers and COPD patients is not enough to claim a higher risk for SARS-CoV-2 infections. The virus’s binding and entrance is the first step of the complex systemic immunological responses in SARS-CoV-2 infections and the activation of the innate immunity through macrophages and DCs, and consequently, T and B cells are also required. The useful effect of nicotine is to reduce cytokine storm mediators via alpha7-nAChRs. nAChrs are involved in many diseases including diseases (diabetes, obesity, cardiovascular, gastrointestinal etc.) which each show similarities with COVID-19 pathogenesis.

Therefore, clever studies are needed to check different doses of nicotine and monitor the background immunological characteristics of COVID-19 patients. Furthermore, all patients should be checked for predisposing factors and possible comorbidities, including cardiovascular diseases, IBD, diabetes, and NAFLD. Therefore, it seems that host factors, the dose of nicotine, and comorbidities determine the harmful or the protective effect of nicotine.

**Fig. 5:**
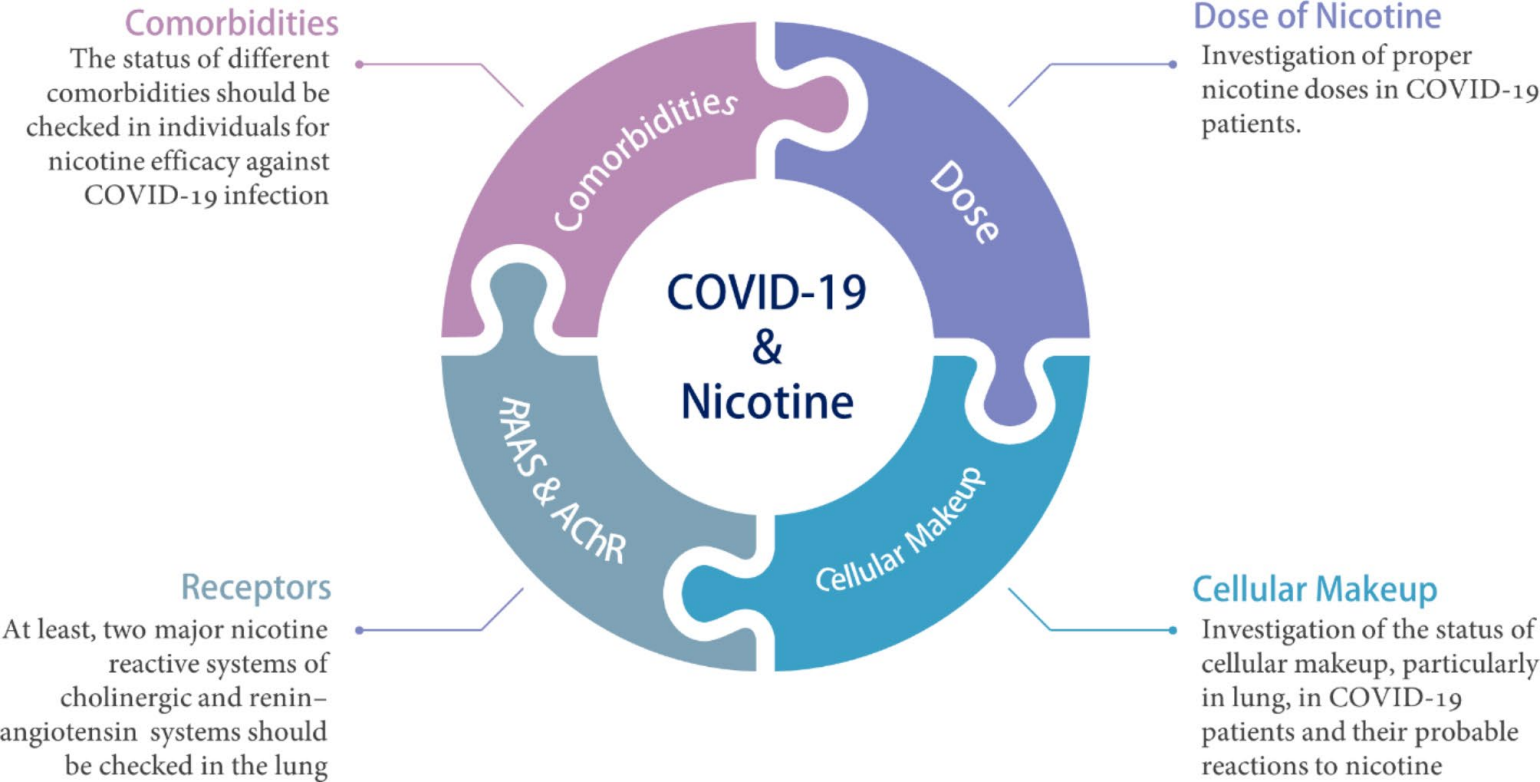
A puzzle diagram indicating the major questions and challenges to conclude the efficacy of nicotine in COVID-19.

As a fundamental approach, we recommend assessing different doses of nicotine on host factors (including inflammatory pathways) to determine what dose, for who and when can be prescribed without any serious harmful effects. The status of macrophages, dendritic, and T and B cells should be also investigated in clinical studies of nicotine’s effect, especially in saliva and sputum samples in COVID-19 patients. The immunopathogenic similarity of COVID-19 and other inflammatory diseases and the effect of nicotine seems to be promising. To prevent the production of erroneous results and its generalization, the effect of smoking should be examined separately from the effect of nicotine. In other words, the results of these two types of studies cannot be easily generalized. Finally, we recommend a simultaneous check-up of RAS and cholinergic systems in nicotine related studies in COVID-19 patients (Fig. 5).

## Data Availability

All data and materials are available thought the corresponding authors upon request.
